# Supplementary material for: Chemical Constituents and Anti-Angiogenic Principles from a Marine Algicolous Penicillium sumatraense SC29
Source: Molecules. 2022 Dec 15;27(24):8940. doi: 10.3390/molecules27248940 (PMC9781389; doi:10.3390/molecules27248940)
Supplement: Supplementary file 1 [file molecules-27-08940-s001.zip › molecules-2042160-supplementary.pdf]

Supporting Information
For
Chemical Constituents and Anti-angiogenesis Principles from a Marine Algicolous *Penicillium steckii* SC29

Hsiao-Yang Hsi, Shih-Wei Wang, Chia-Hsiung Cheng, Ka-Lai Pang, Jyh-Yih Leu, Szu-Hsing Chang, Yen-Tung Lee, Yueh-Hsiung Kuo, Chia-Ying Huang and Tzong-Huei Lee

Page 3, Figure S1. ^{1}H NMR (600 MHz, MeOH-d_4) of **1**.
Figure S2. ^{13}C NMR (150 MHz, MeOH-d_4) of **1**.
Page 4, Figure S3. HSQC of **1**.
Figure S4. COSY of **1**.
Page 5, Figure S5. HMBC of **1**.
Figure S6. IR spectrum of **1**.
Page 6, Figure S7. HRESIMS spectrum of **1**.
Page 7, Figure S8. ^{1}H NMR (600 MHz, MeOH-d_4) of **2**.
Figure S9. ^{13}C NMR (150 MHz, MeOH-d_4) of **2**.
Page 8, Figure S10. HSQC of **2**.
Figure S11. COSY of **2**.
Page 9, Figure S12. HMBC of **2**.
Figure S13. IR spectrum of **2**.
Page 10, Figure S14. HRESIMS spectrum of **2**.
Page 11, Figure S15. ^{1}H NMR (600 MHz, MeOH-d_4) of **3**.
Figure S16. ^{13}C NMR (150 MHz, MeOH-d_4) of **3**
Page 12, Figure S17. HSQC of **3**.
Figure S18. COSY of **3**.
Page 13, Figure S19. HMBC of **3**.
Figure S20. NOESY of **3**.
Page 14, Figure S21. IR spectrum of **3**.
Figure S22. HRESIMS spectrum of **3**.
Page 15, Figure S23. ^{1}H NMR (600 MHz, MeOH-d_4) of **4**.
Figure S24. ^{13}C NMR (150 MHz, MeOH-d_4) of **4**.
Page 16, Figure S25. HSQC of **4**.
Figure S26. COSY of **4**.
Page 17, Figure S27. HMBC of **4**.
Figure S28. IR spectrum of **4**.
Page 18, Figure S29. HRESIMS spectrum of **4**
Page 19, Figure S30. ^{1}H NMR (600 MHz, MeOH-d_4) of **5**.

Figure S31. ^{13}C NMR (150 MHz, MeOH-d_4) of **5**.
Page 20, Figure S32. HSQC of **5**.
Figure S33. COSY of **5**.
Page 21, Figure S34. HMBC of **5**.
Figure S35. NOESY of **5**.
Page 22, Figure S36. IR spectrum of **5**.
Figure S37. HRESIMS spectrum of **5**.
Page 23, Figure S38. ^{1}H NMR (600 MHz, MeOH-d_4) of **6**.
Figure S39. ^{13}C NMR (150 MHz, MeOH-d_4) of **6**.
Page 24, Figure S40. Effects of compounds **1**, **2**, **3**, and **5** on tube formation of human endothelial progenitor cells
Page 25, Figure S41. Effects of compounds **1**-**5** on cell migration of human endothelial progenitor cells.
Page 26, Figure S42. Effect of compound **4** on the apoptotic cell death of human endothelial progenitor cells.
Page 27, Figure S43. Antiangiogenesis of compound **1**, **2**, and **5** using transgenic zebrafish.
Page 28, Figure S44. ITS rDNA sequences.
Page 29, Figure S45. Agarose gel electrophoresis.
Page 30, Figure S46. BLASTn results of the isolated fungus.
Page 31, Table S1. Crystal data and experimental details for **5**.
Page 32, Table S2. Atomic coordinates (x 10^4) and equivalent isotropic displacement parameters (Å^2 x 10^3)
Page 33, Table S3. Bond lengths [Å] and angles [°] for **5**.
Page 35, Table S4. Anisotropic displacement parameters (Å^2 x 10^3) for **5**.

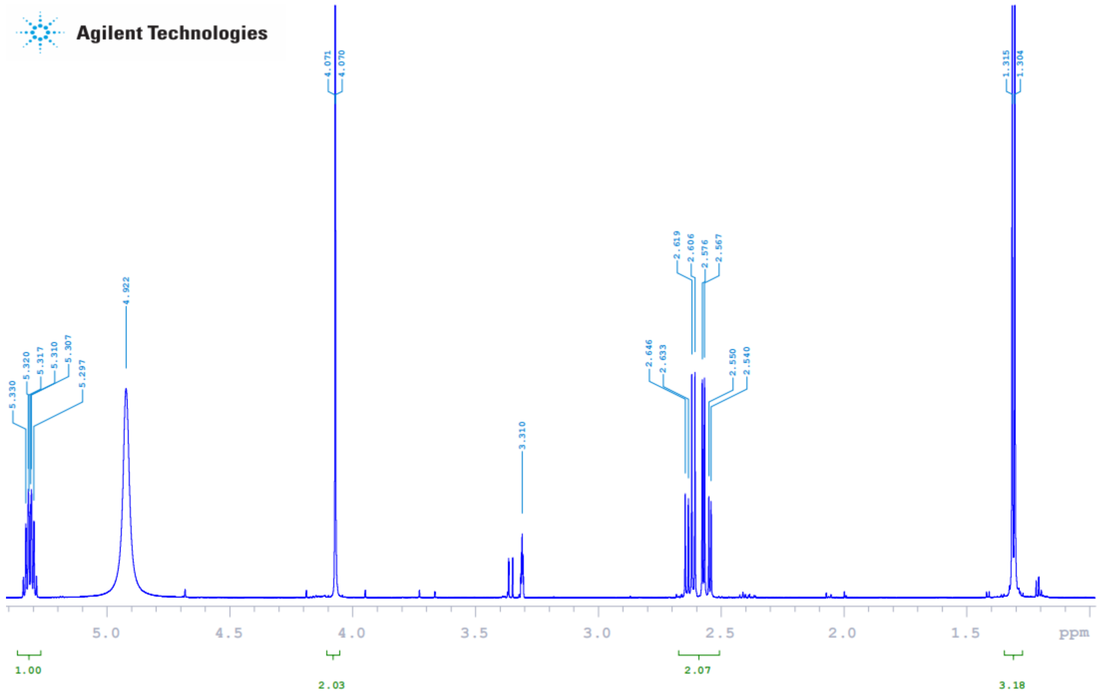

Figure S1. ^{1}H NMR (600 MHz, MeOH-d_4) of **1**.

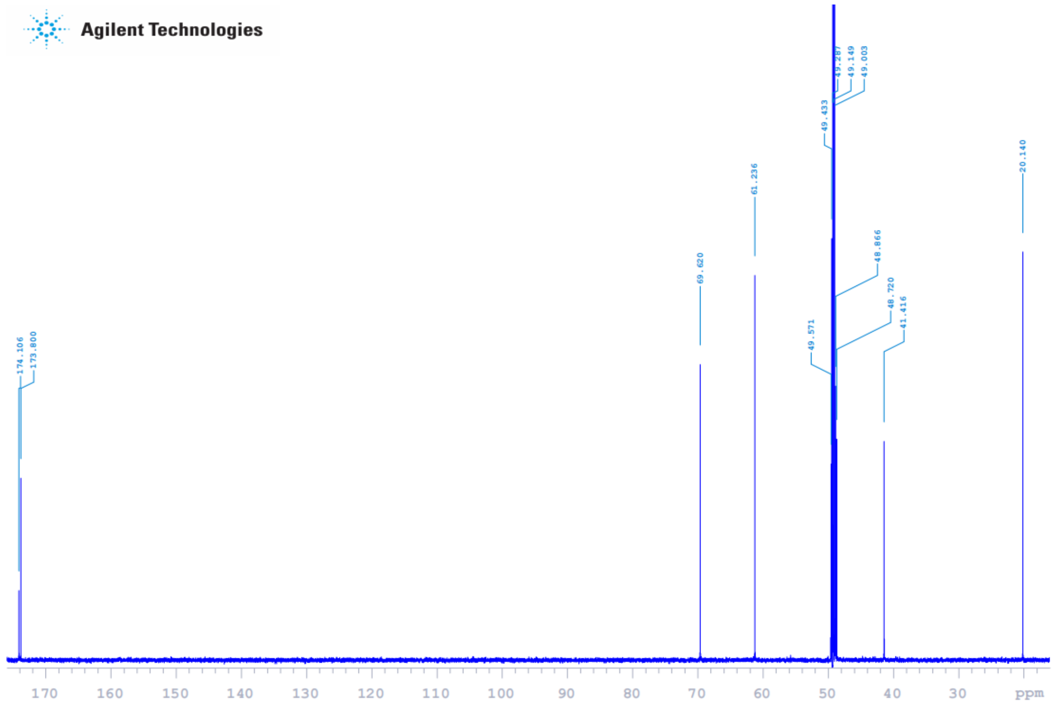

Figure S2. ^{13}C NMR (150 MHz, MeOH-d_4) of **1**.

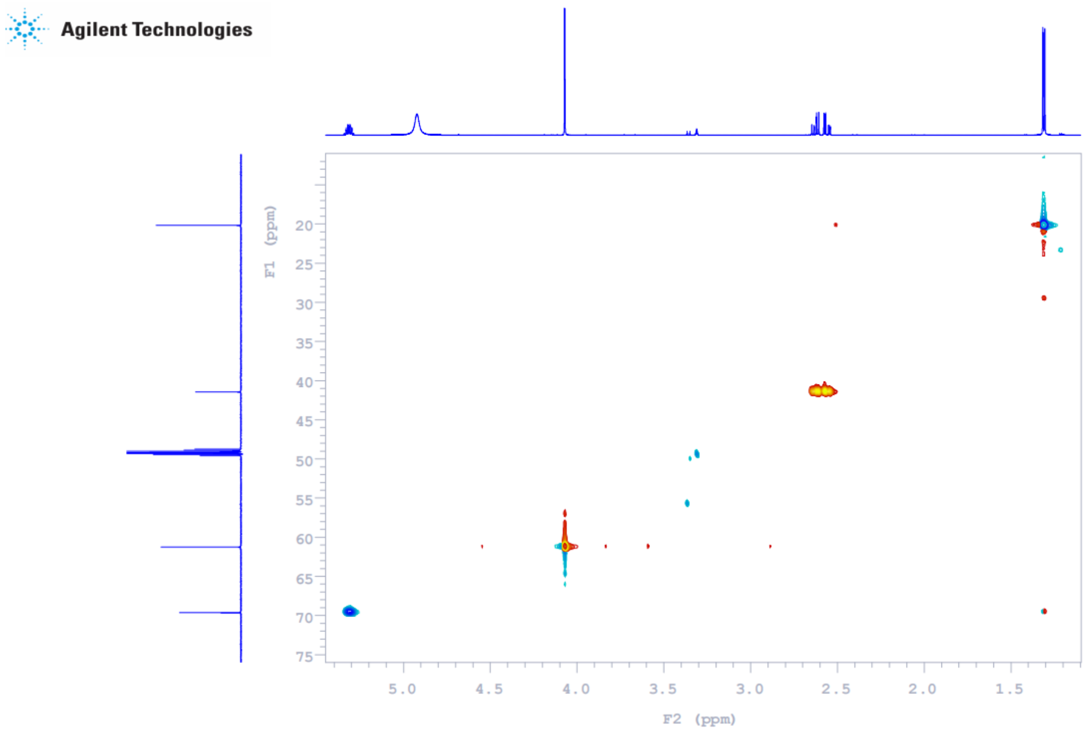

Figure S3. HSQC of **1**.

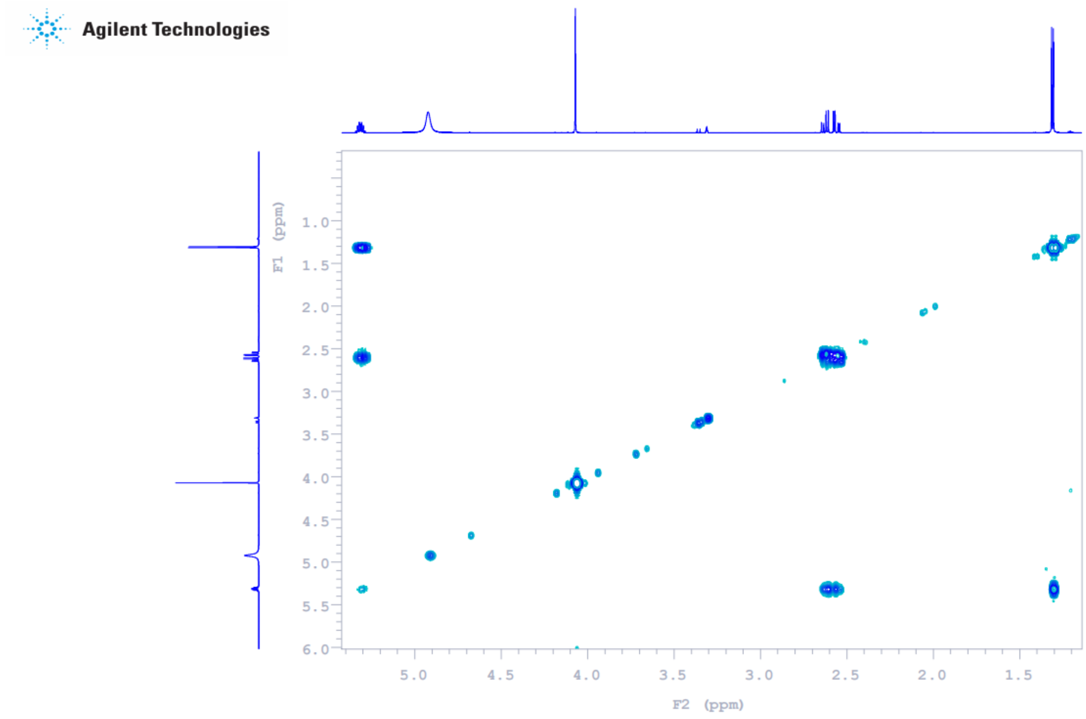

Figure S4. COSY of **1**.

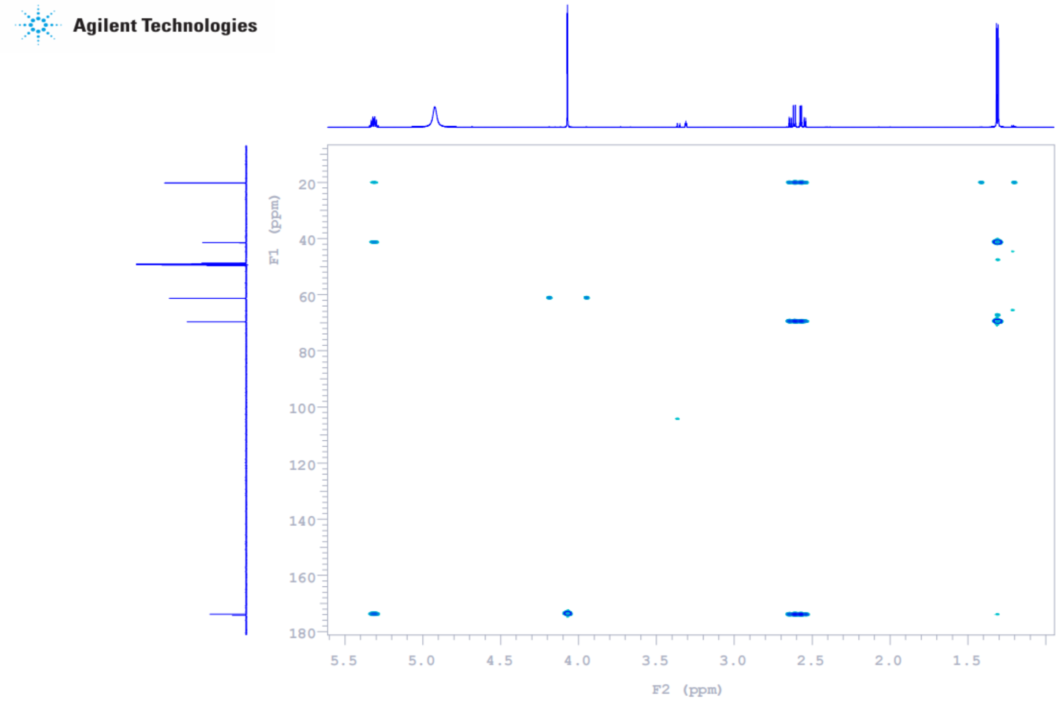

Figure S5. HMBC of **1**.

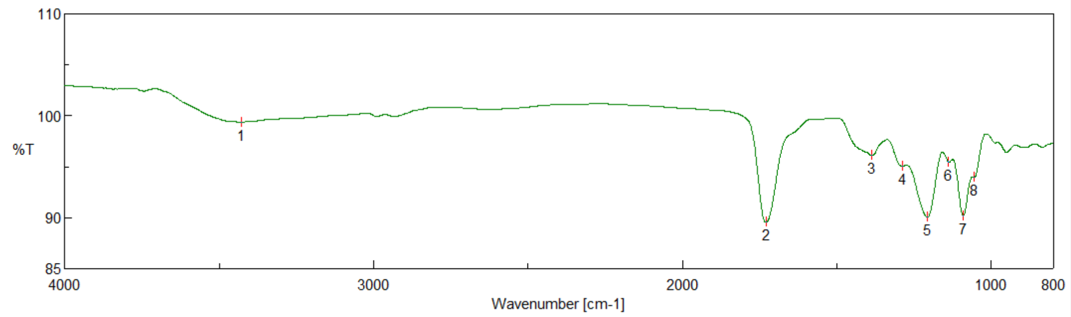

[Result of Peak Picking]

No.	Position	Intensity	No.	Position	Intensity	No.	Position	Intensity	No.	Position	Intensity
1	3426.89	99.3472	2	1728.87	89.5271	3	1387.53	96.0617	4	1288.22	95.0469
5	1207.22	90.0175	6	1138.76	95.4686	7	1090.55	90.1544	8	1055.84	93.9669

Figure S6. IR spectrum of **1**.

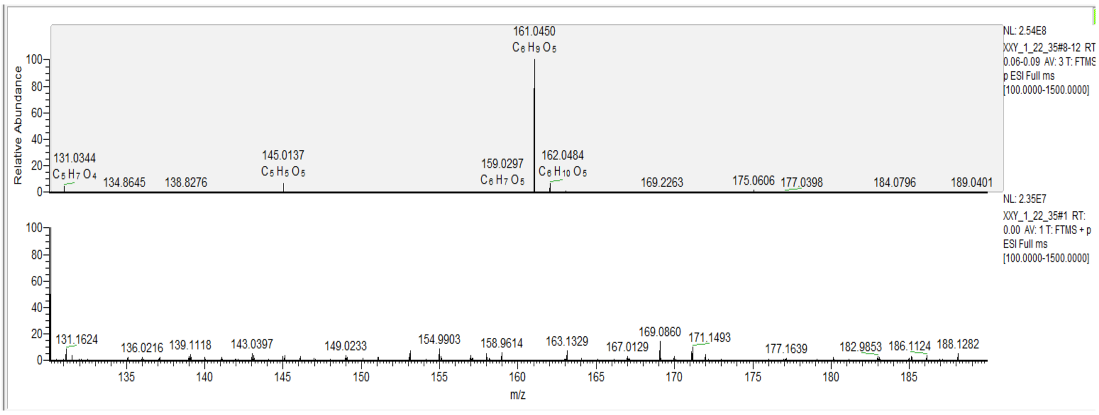

Figure S7. HRESIMS spectrum of **1**.

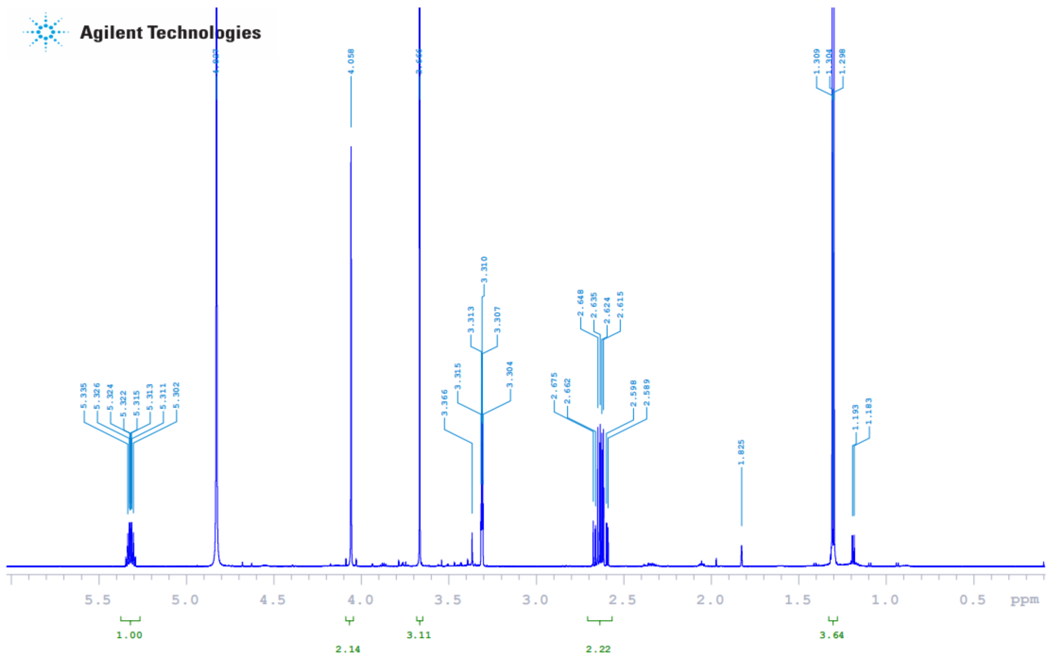

Figure S8. ^{1}H NMR (600 MHz, MeOH-d_4) of **2**.

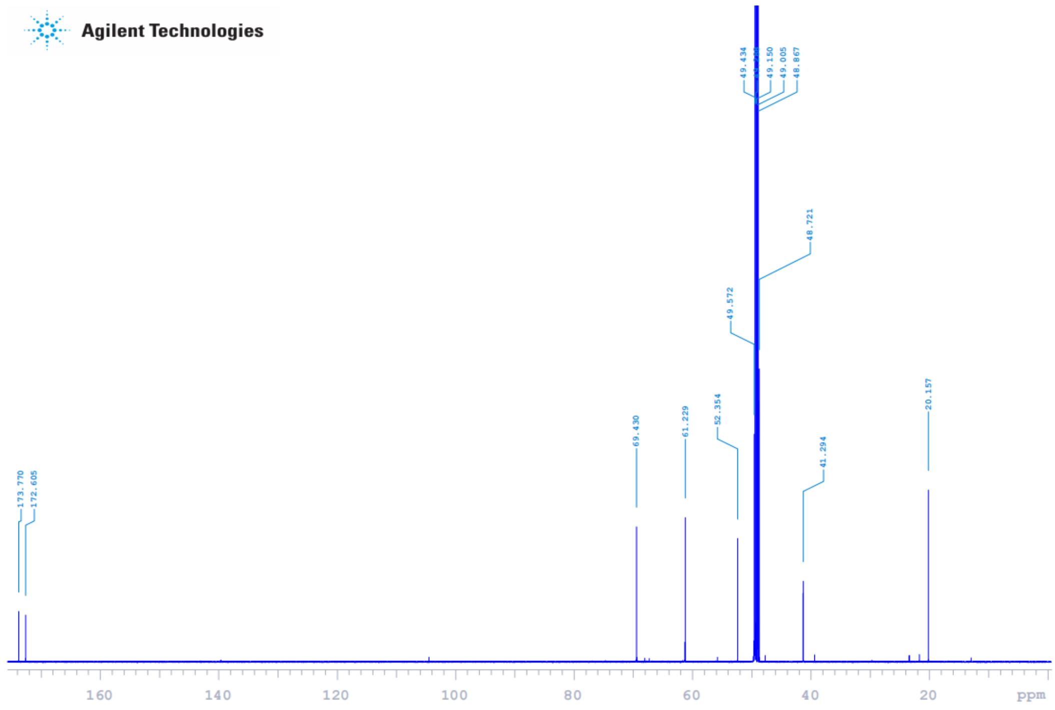

Figure S9. ^{13}C NMR (150 MHz, MeOH-d_4) of **2**.

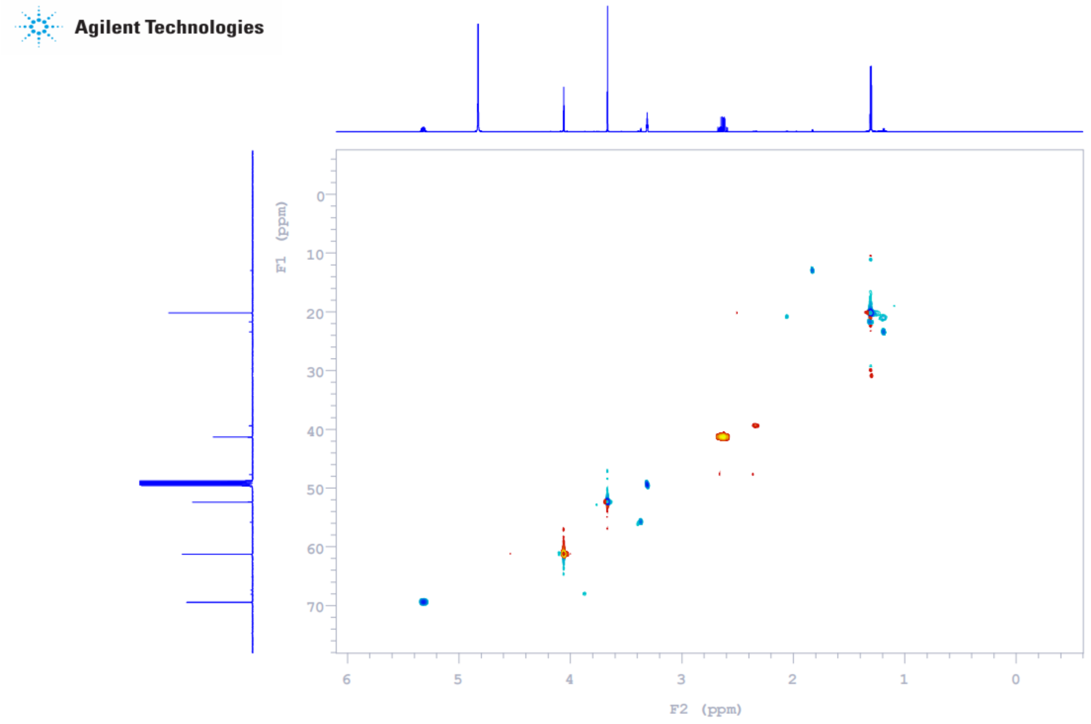

Figure S10. HSQC of **2**.

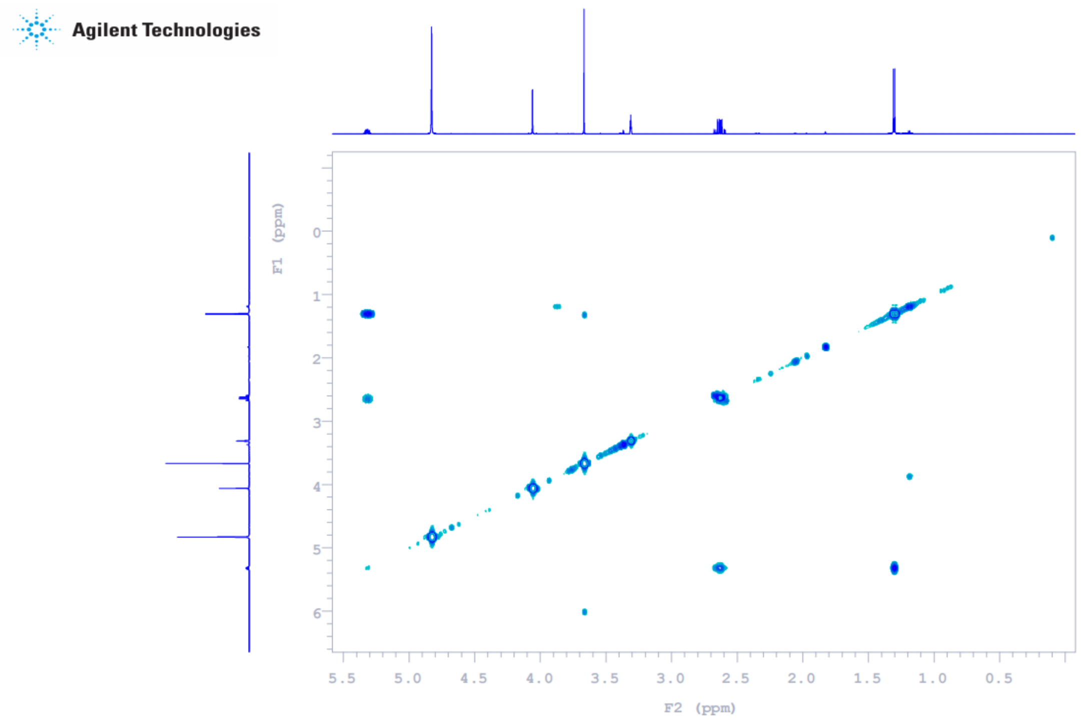

Figure S11. COSY of **2**.

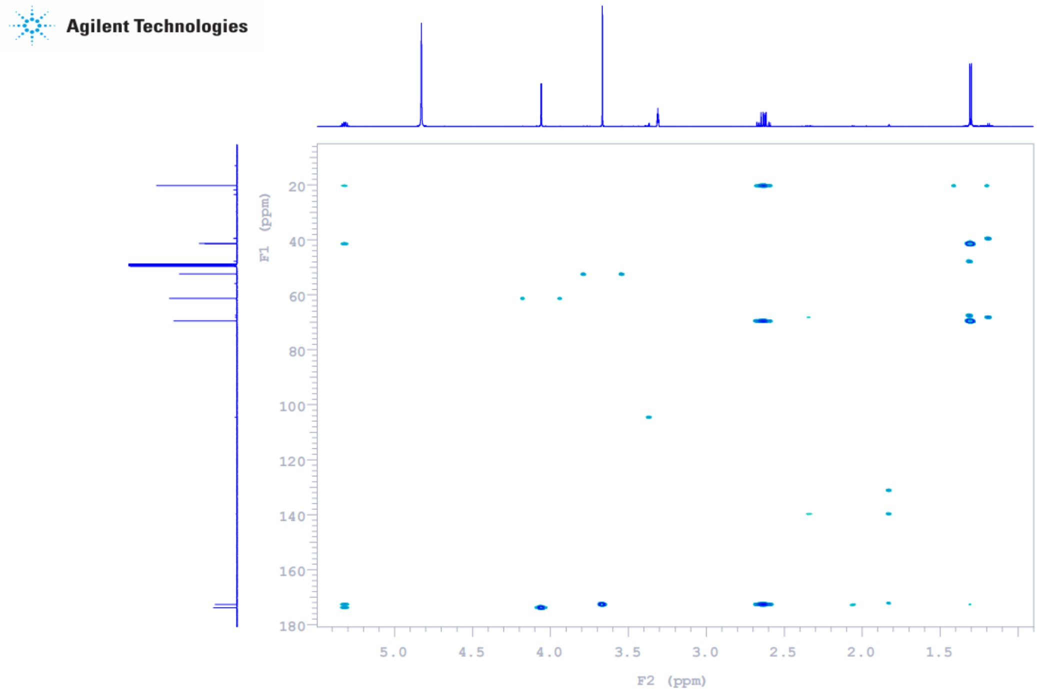

Figure S12. HMBC of **2**.

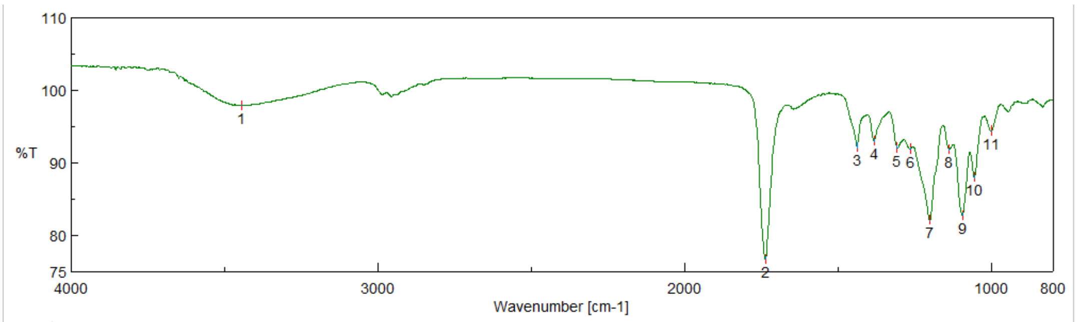

[Result of Peak Picking]

No.	Position	Intensity	No.	Position	Intensity	No.	Position	Intensity	No.	Position	Intensity
1	3445.21	97.9032	2	1737.55	76.7691	3	1439.6	92.1791	4	1384.64	93.0155
5	1308.46	92.078	6	1266.04	91.9431	7	1201.43	82.1295	8	1138.76	91.8496
9	1096.33	82.7827	10	1055.84	88.0668	11	1001.84	94.3939			

Figure S13. IR spectrum of **2**.

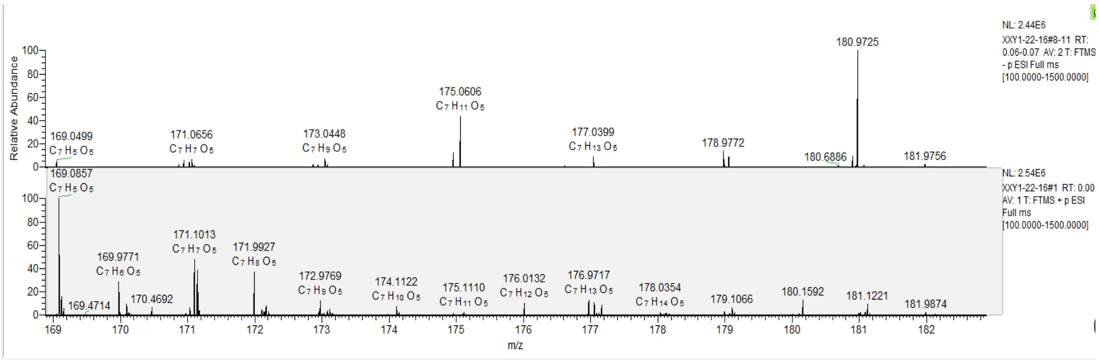

Figure S14. HRESIMS spectrum of **2**.

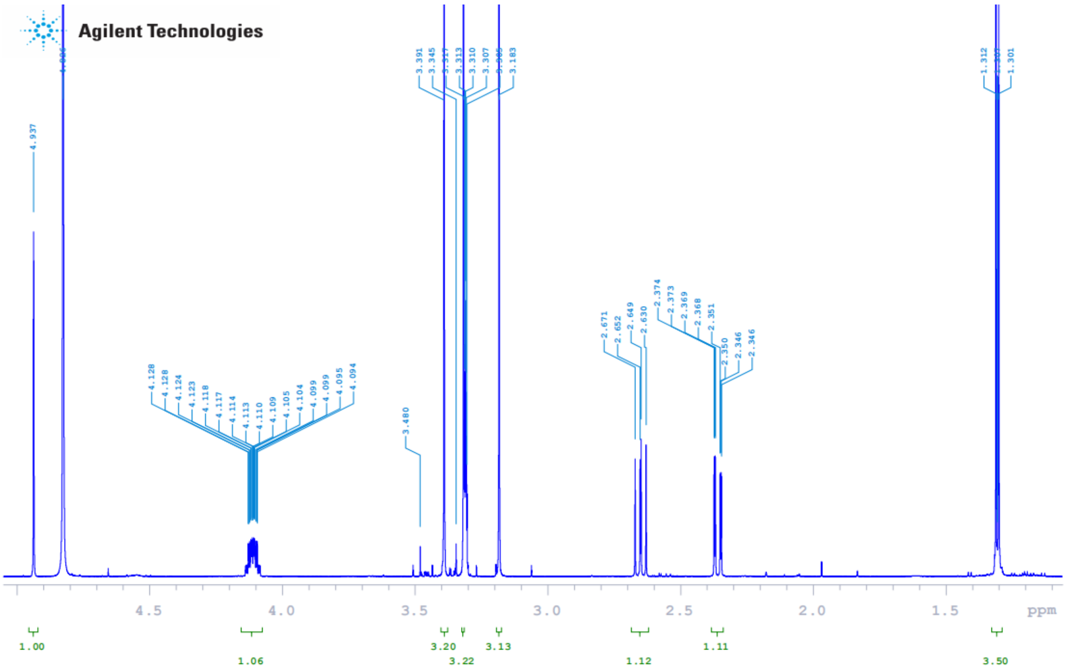

Figure S15. ^{1}H NMR (600 MHz, MeOH-d_4) of **3**.

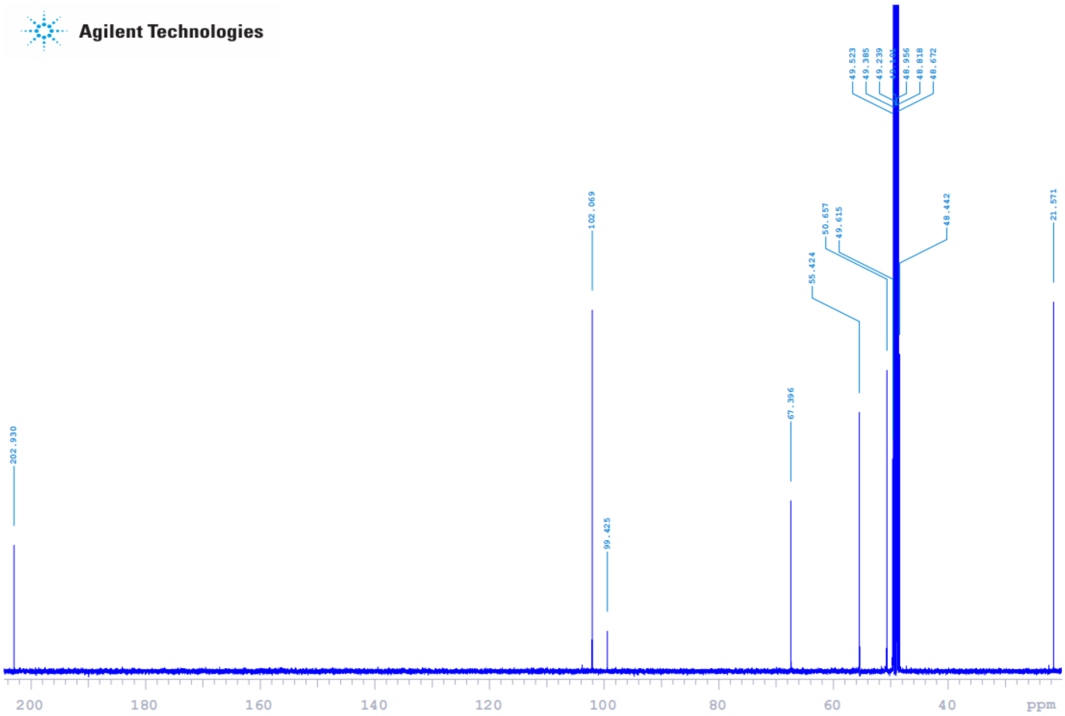

Figure S16. ^{13}C NMR (150 MHz, MeOH-d_4) of **3**.

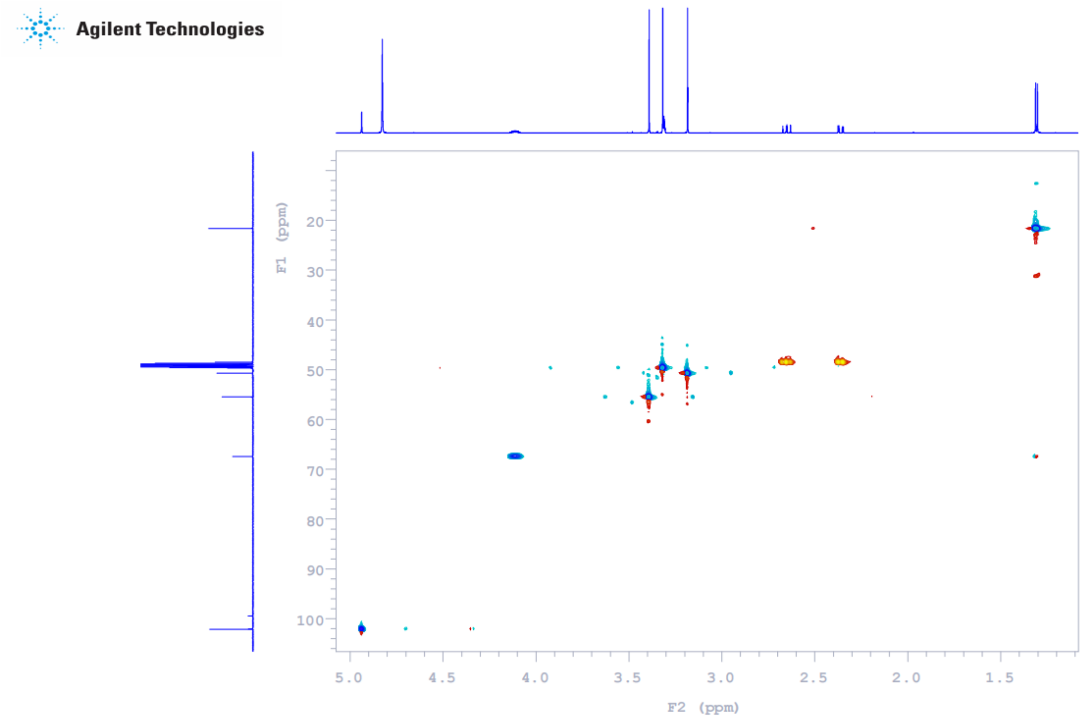

Figure S17. HSQC of **3**.

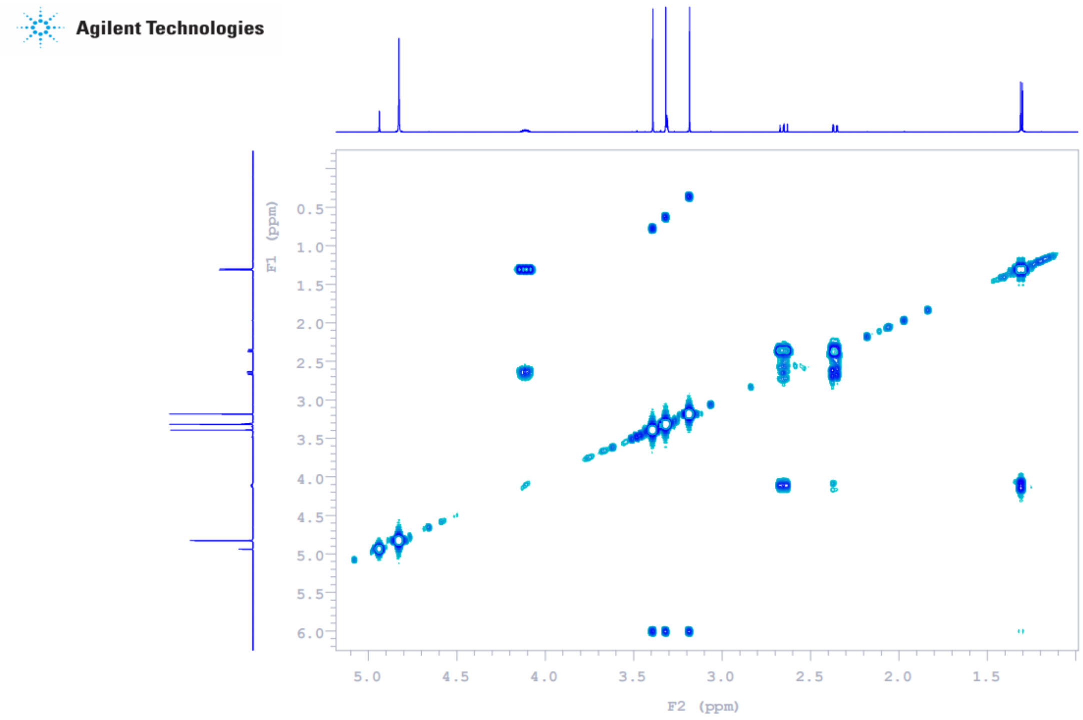

Figure S18. COSY of **3**.

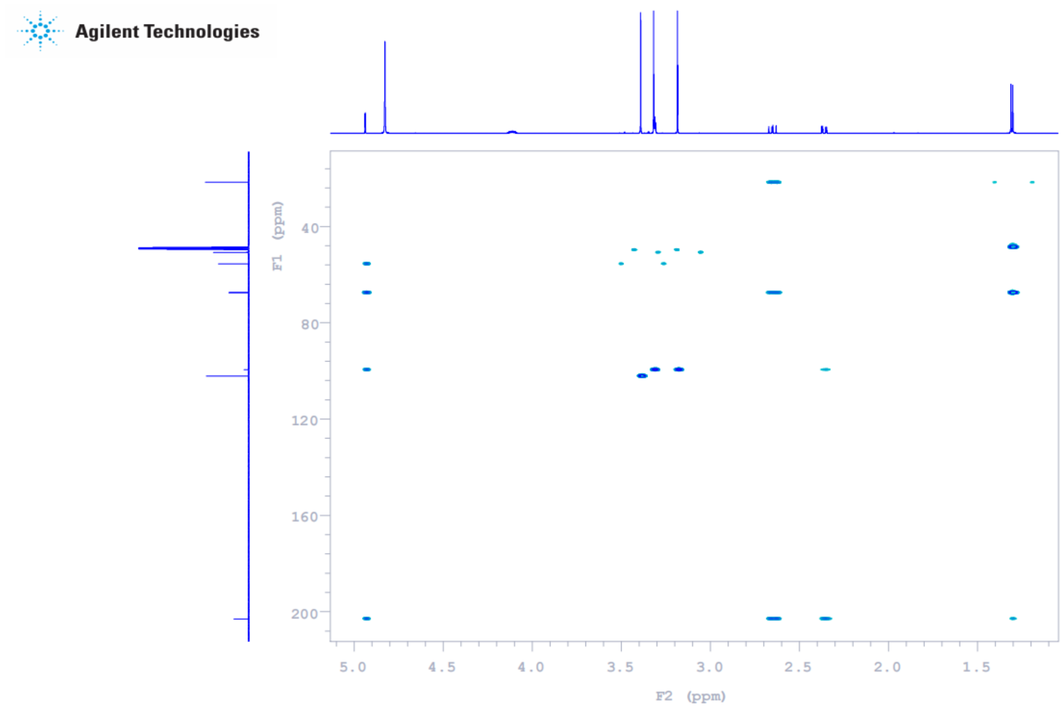

Figure S19. HMBC of **3**.

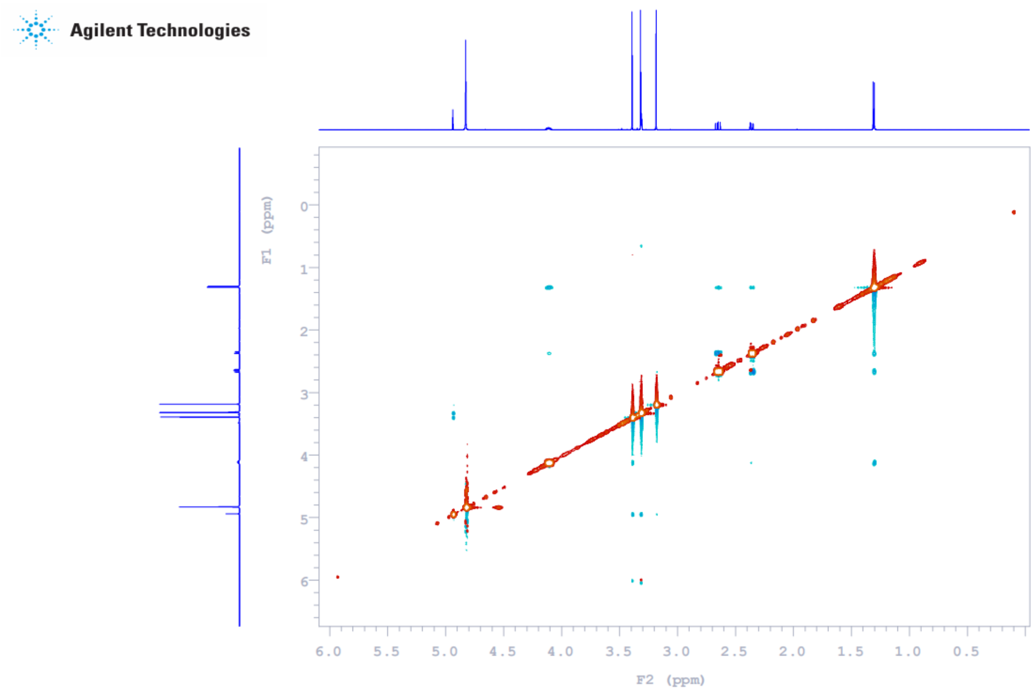

Figure S20. NOESY of **3**.

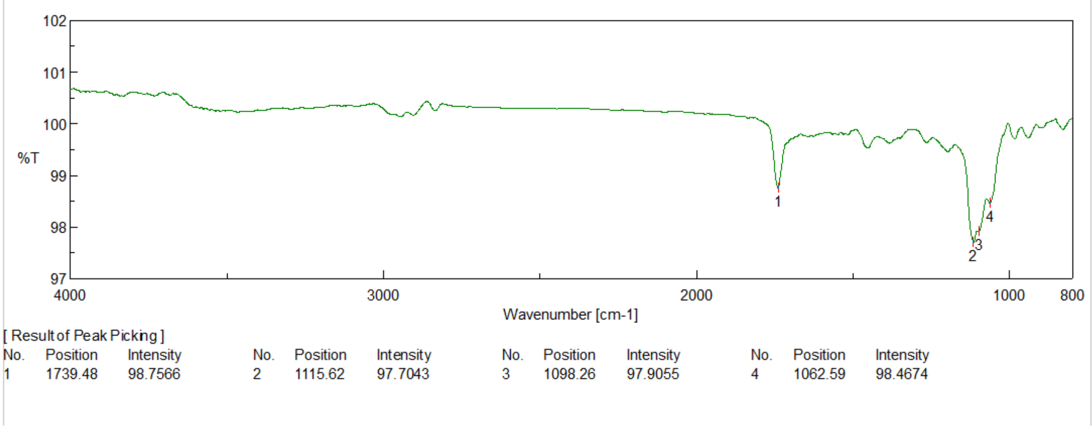

Figure S21. IR spectrum of **3**.

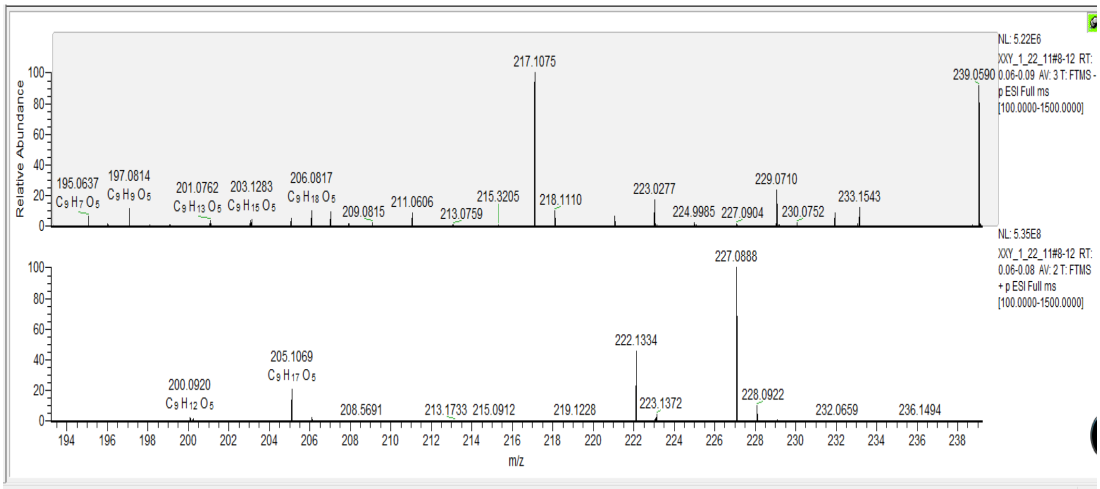

Figure S22. HRESIMS spectrum of **3**.

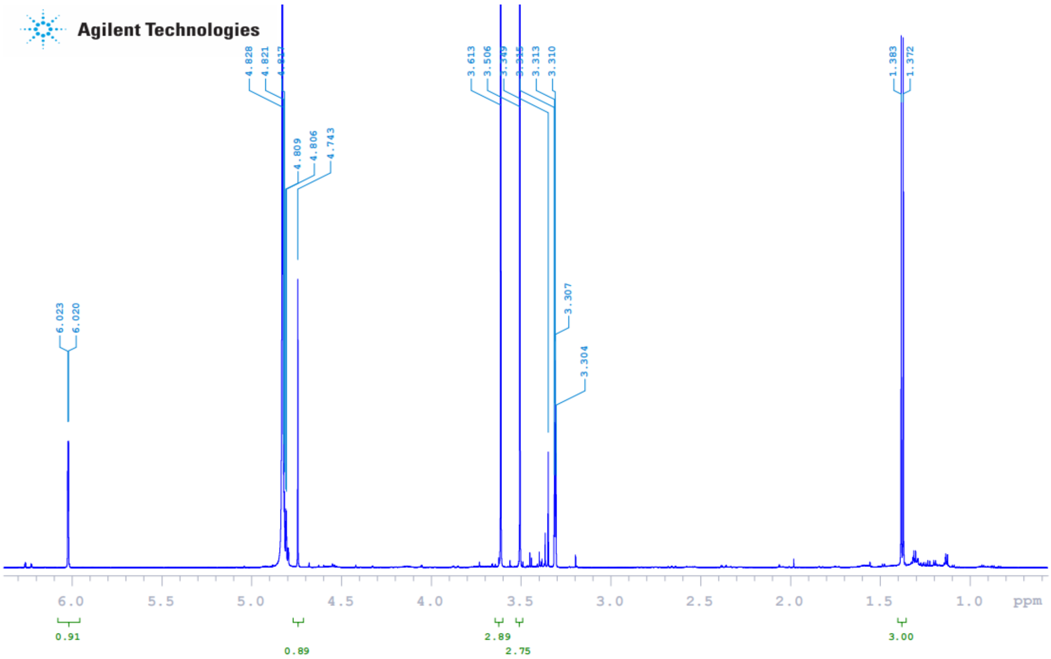

Figure S23. ^{1}H NMR (600 MHz, MeOH-d_4) of **4**.

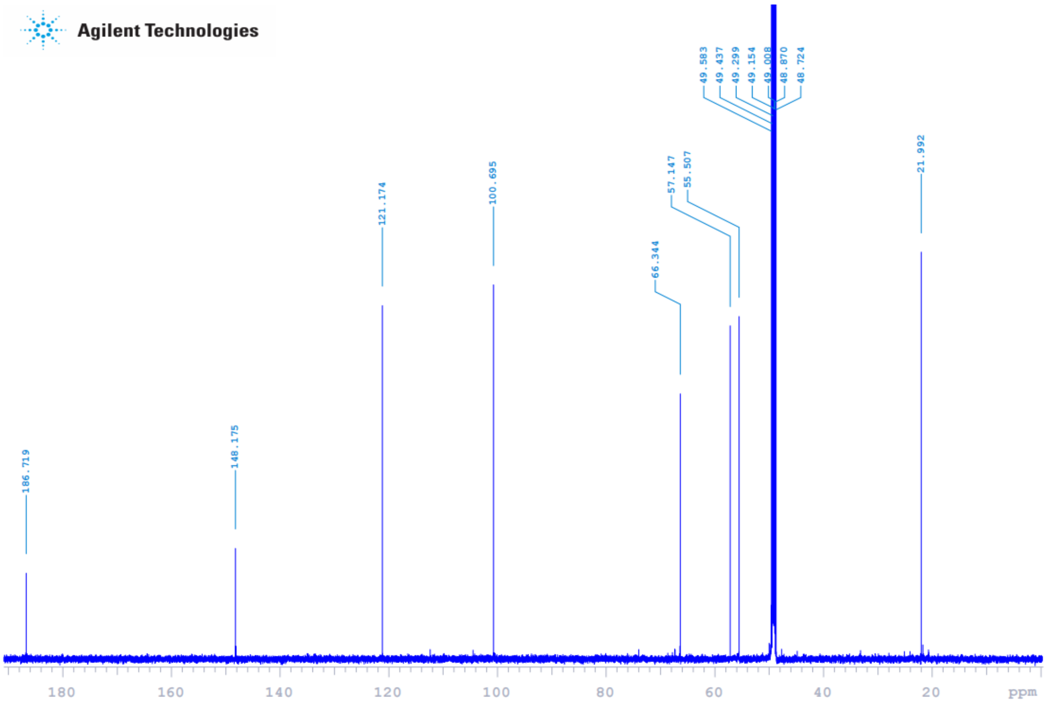

Figure S24. ^{13}C NMR (150 MHz, MeOH-d_4) of **4**.

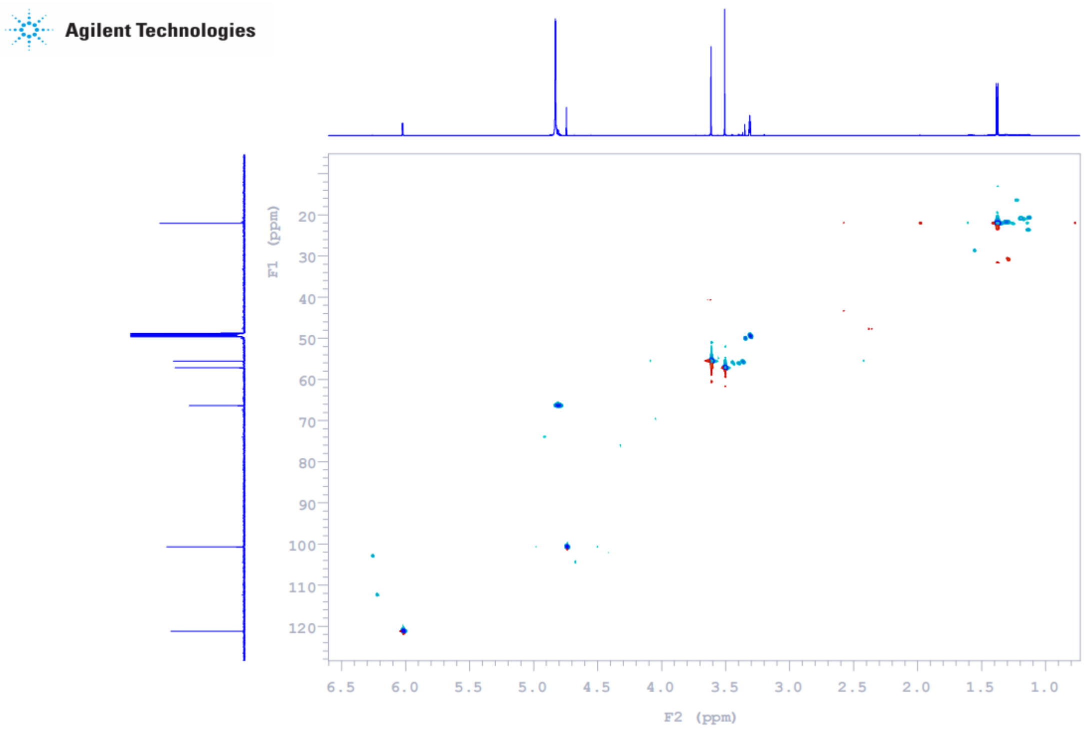

Figure S25. HSQC of **4**.

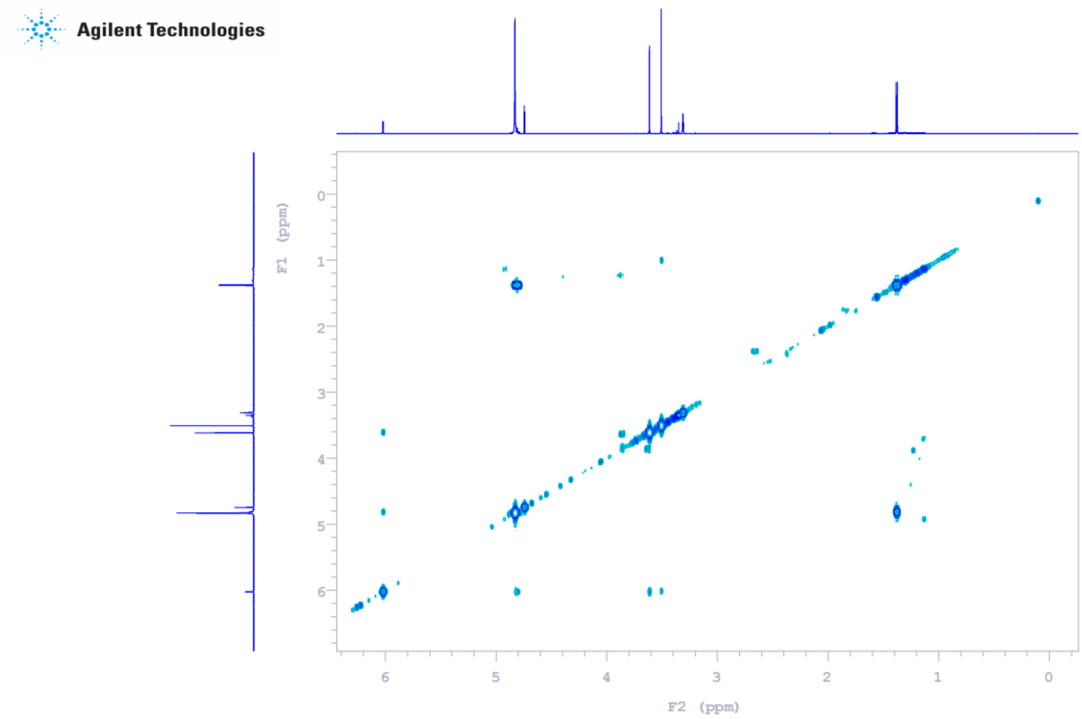

Figure S26. COSY of **4**.

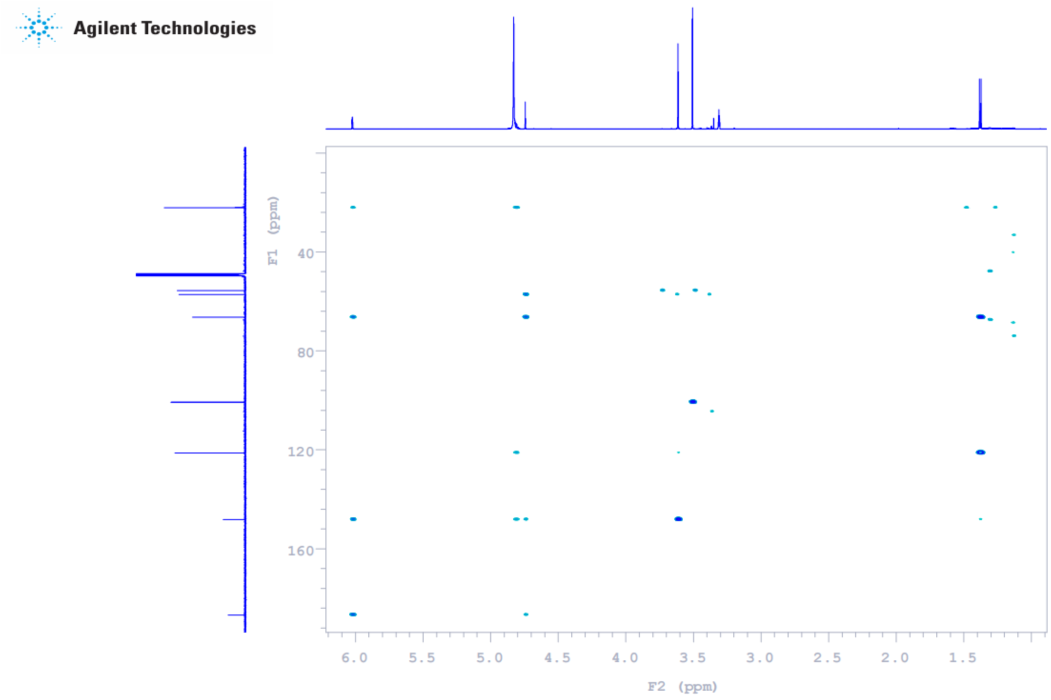

Figure S27. HMBC of **4**.

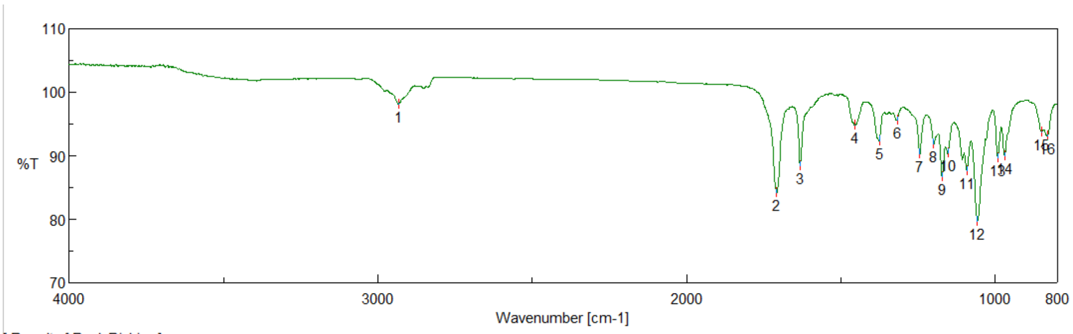

[Result of Peak Picking]

No.	Position	Intensity	No.	Position	Intensity	No.	Position	Intensity	No.	Position	Intensity
1	2933.2	98.1087	2	1707.66	84.1529	3	1632.45	88.4106	4	1455.99	94.7776
5	1375.96	92.2692	6	1318.11	95.579	7	1244.83	90.3039	8	1199.51	91.8393
9	1172.51	86.7106	10	1153.22	90.4129	11	1091.51	87.5693	12	1057.76	79.642
13	992.196	89.6316	14	970.019	89.9227	15	848.525	93.7465	16	832.133	93.017

Figure S28. IR spectrum of **4**.

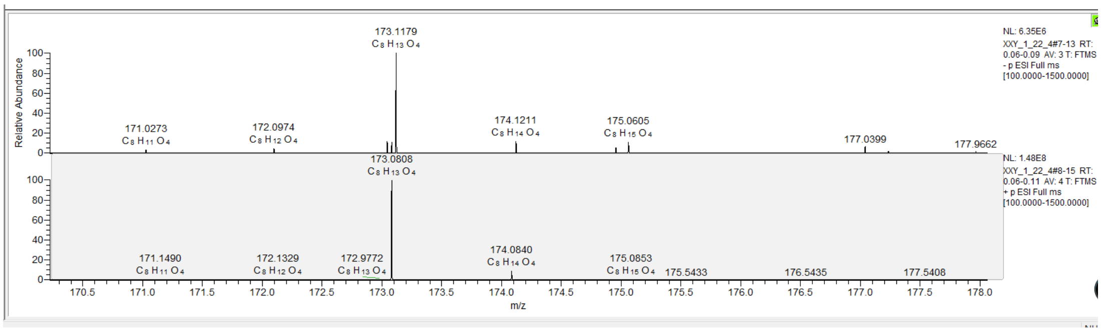

Figure S29. HRESIMS spectrum of **4**.

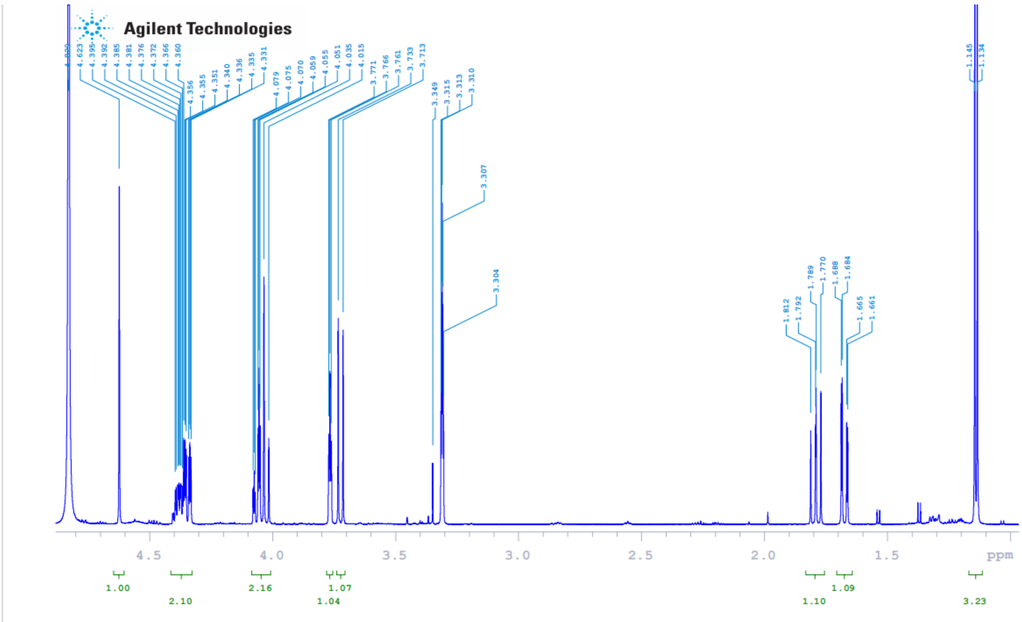

Figure S30. ^{1}H NMR (600 MHz, MeOH-d_4) of **5**.

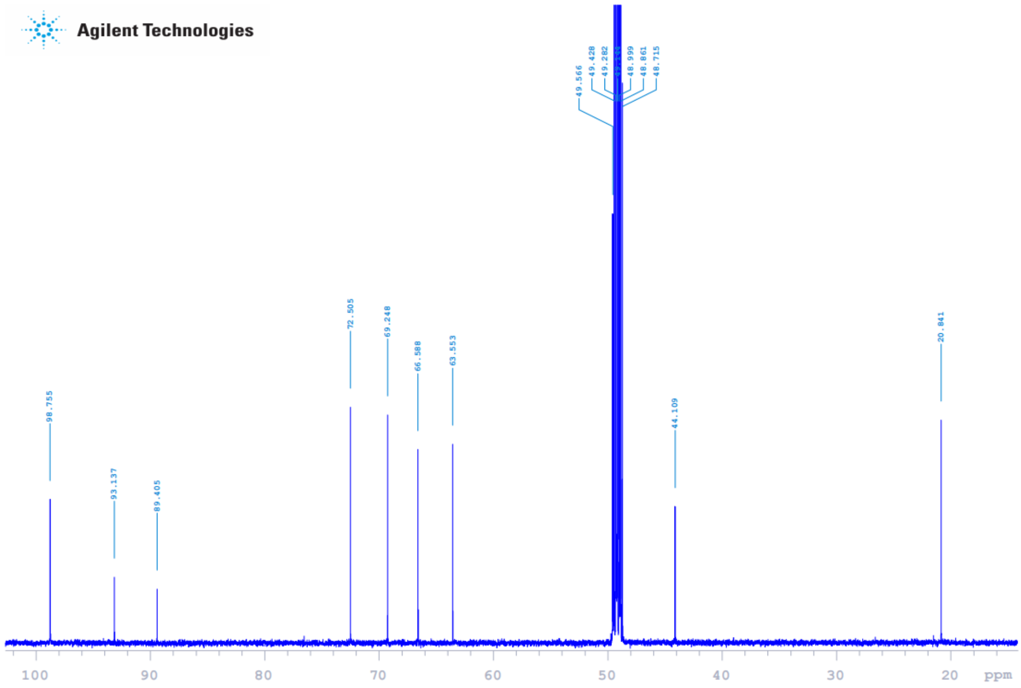

Figure S31. ^{13}C NMR (150 MHz, MeOH-d_4) of **5**.

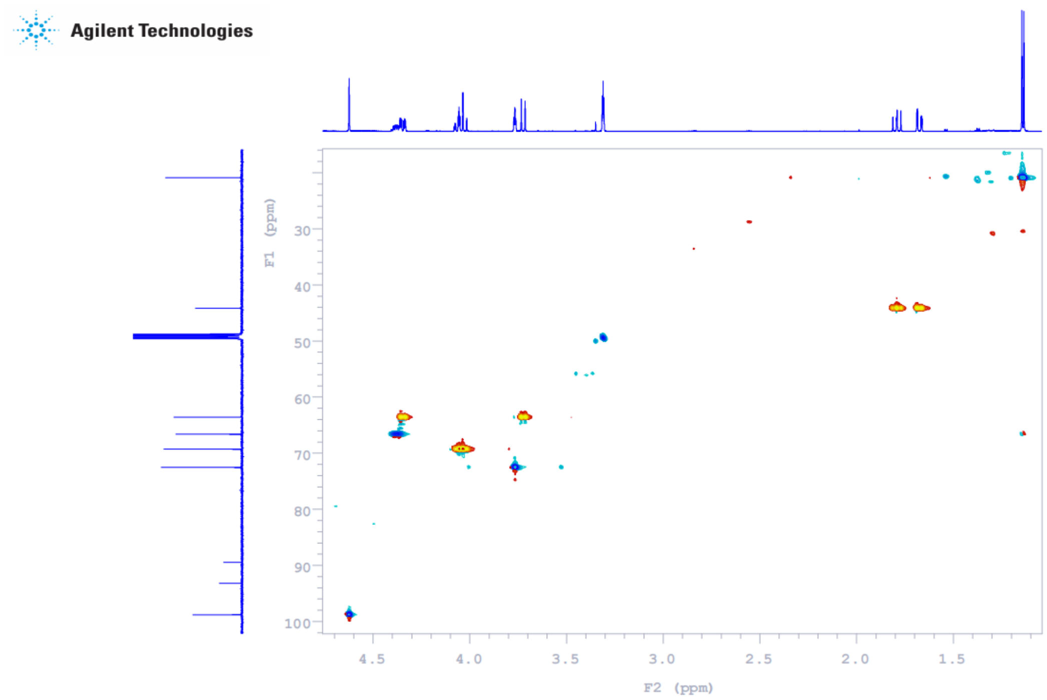

Figure S32. HSQC of **5**.

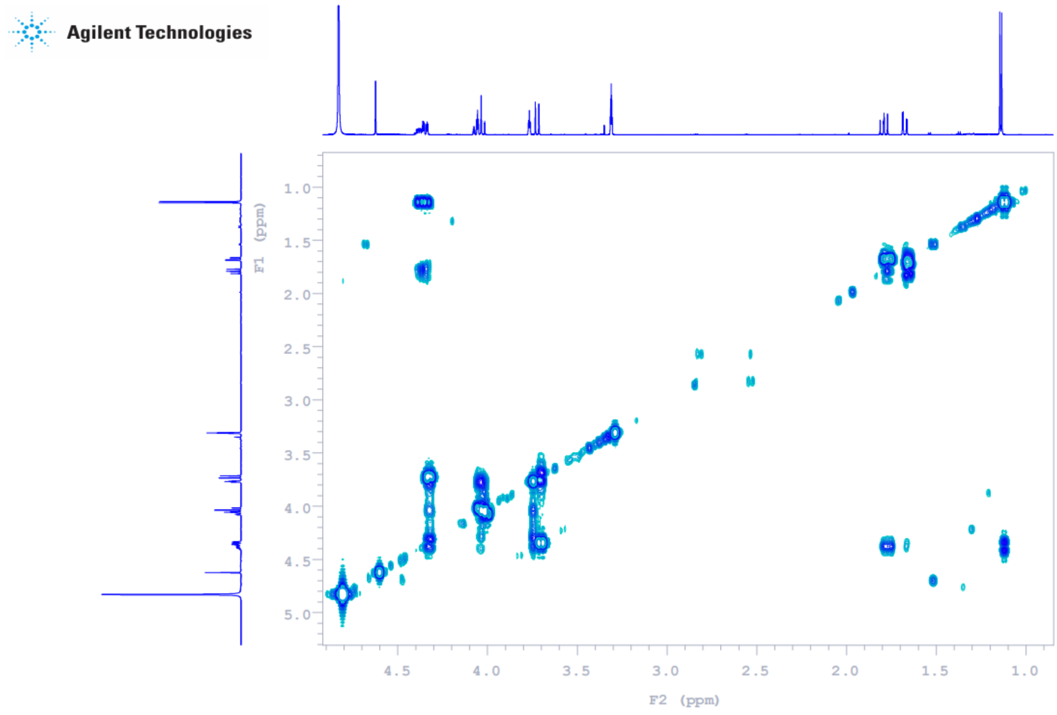

Figure S33. COSY of **5**.

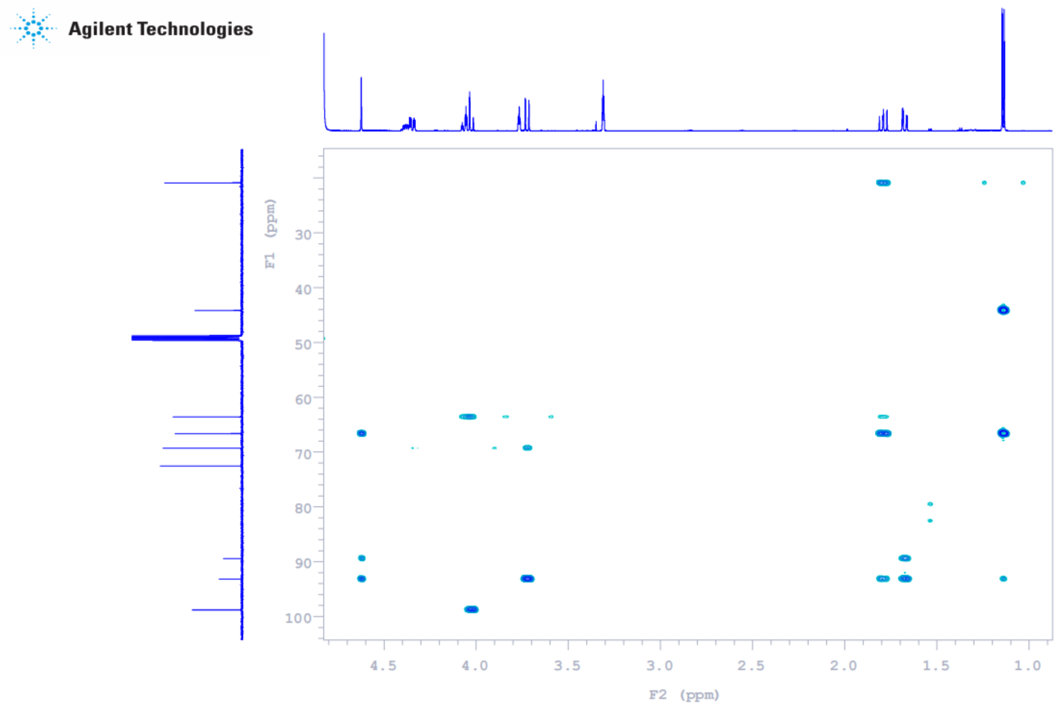

Figure S34. HMBC of **5**.

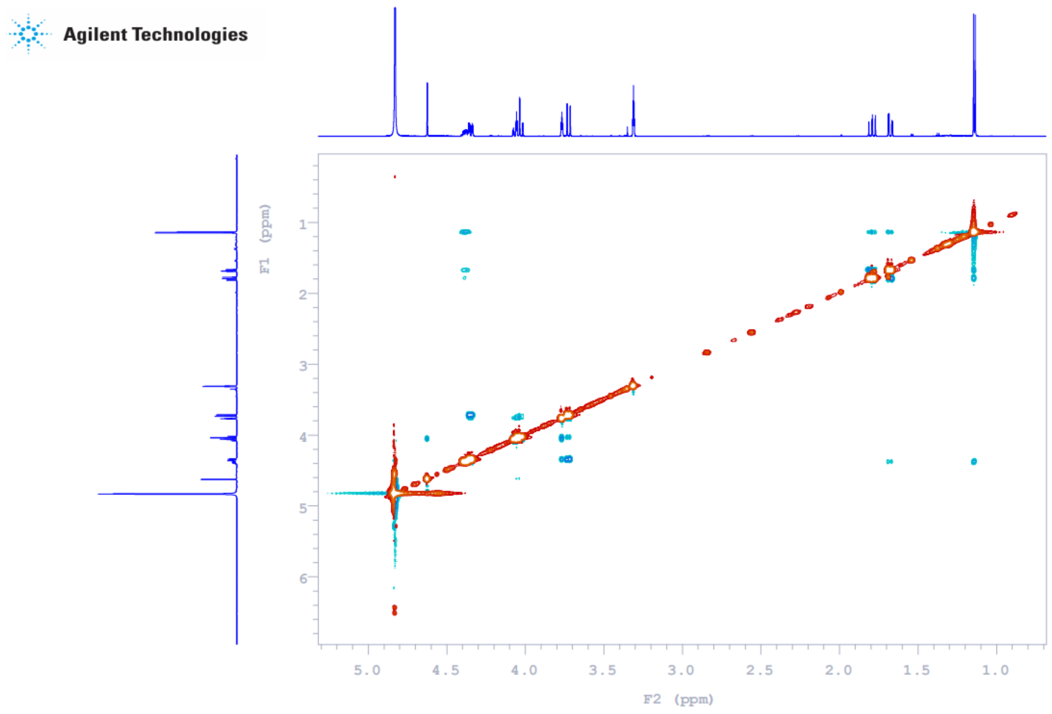

Figure S35. NOESY of **5**.

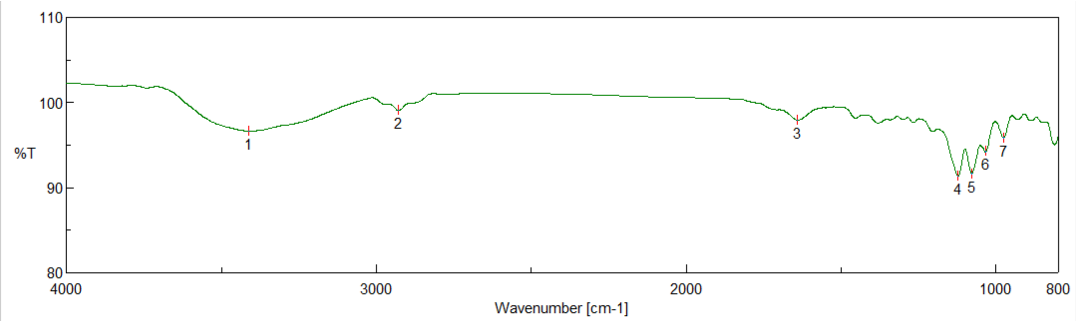

[Result of Peak Picking]

No.	Position	Intensity	No.	Position	Intensity	No.	Position	Intensity	No.	Position	Intensity
1	3411.46	96.6157	2	2928.38	99.0224	3	1641.13	97.8983	4	1122.37	91.3254
5	1078.01	91.6063	6	1031.73	94.217	7	975.804	95.8341			

Figure S36. IR spectrum of **5**.

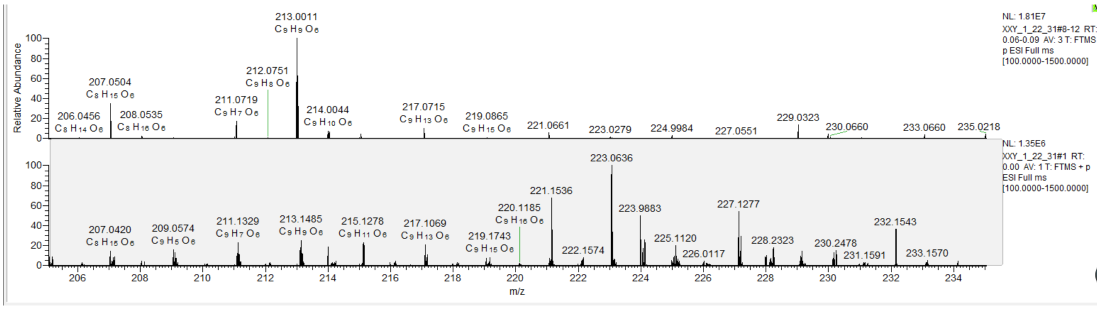

Figure S37. HRESIMS spectrum of **5**.

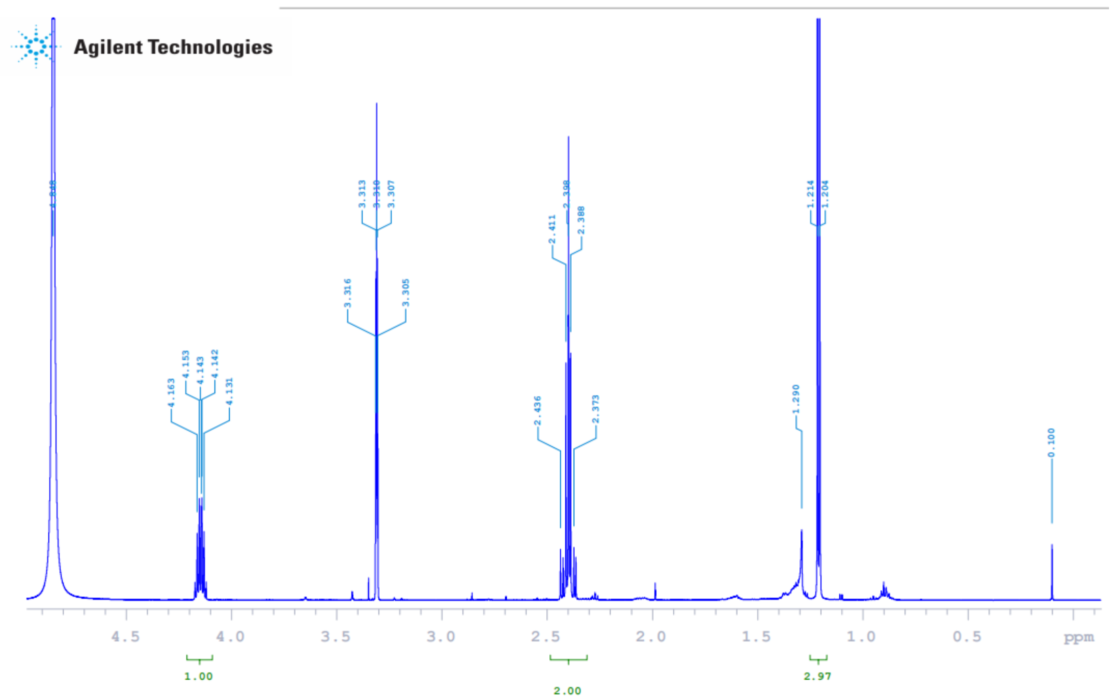

Figure S38. ^{1}H NMR (600 MHz, MeOH-d_4) of **6**.

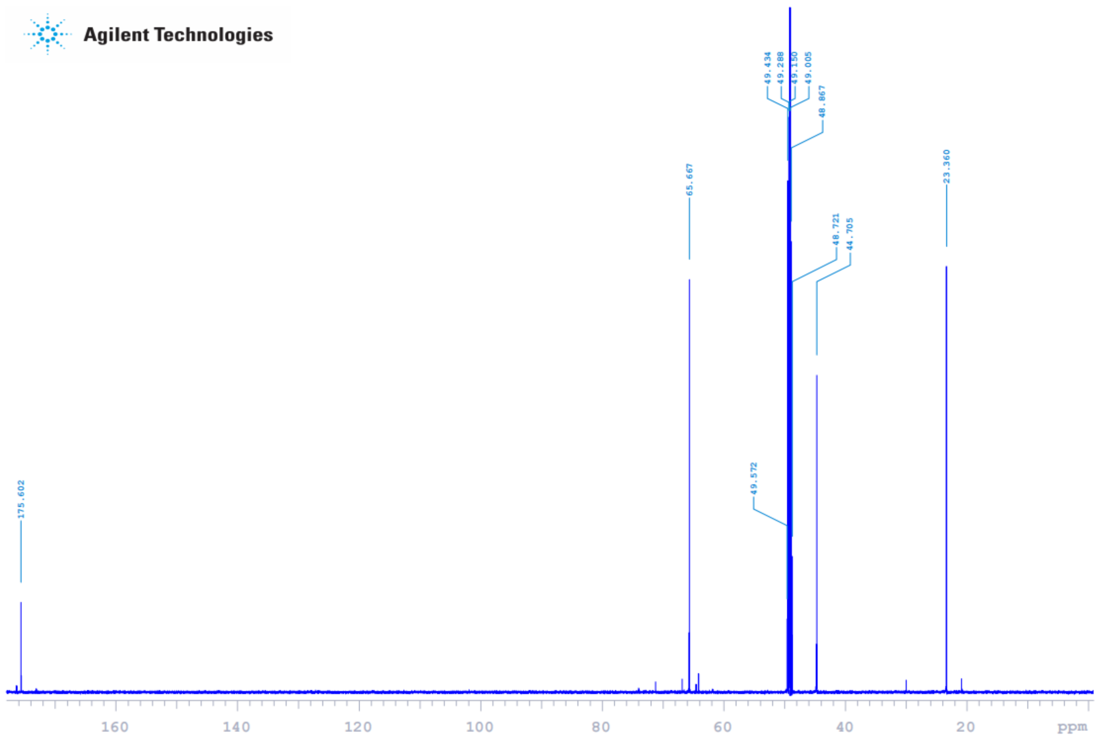

Figure S39. ^{13}C NMR (150 MHz, MeOH-d_4) of **6**.

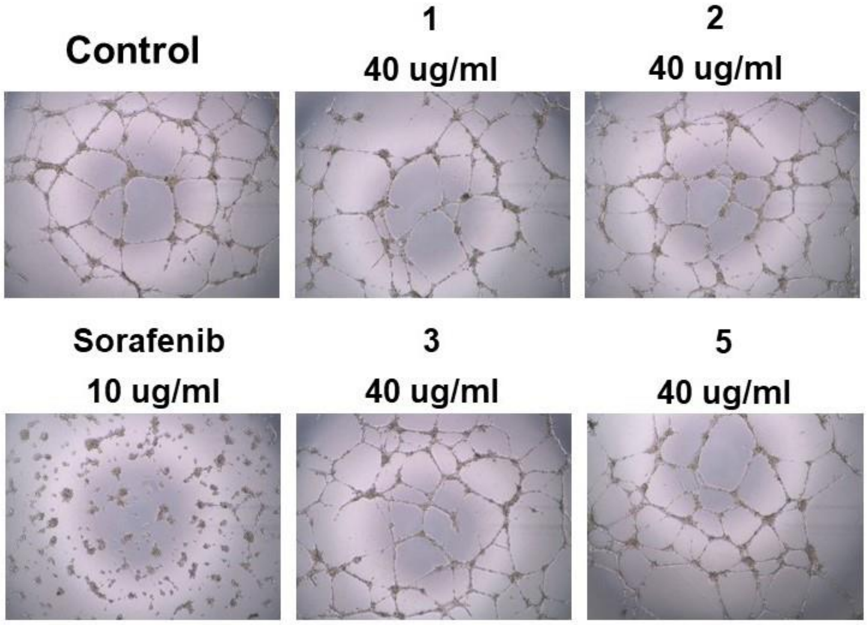

Figure S40. Effects of compounds 1, 2, 3, and 5 on tube formation of human endothelial progenitor cells. Cells were treated with the indicated compounds for 24 h. The capillary-like structure formation was examined by tube formation assay. Data represent the mean ± S.E.M. of 3 independent experiments.

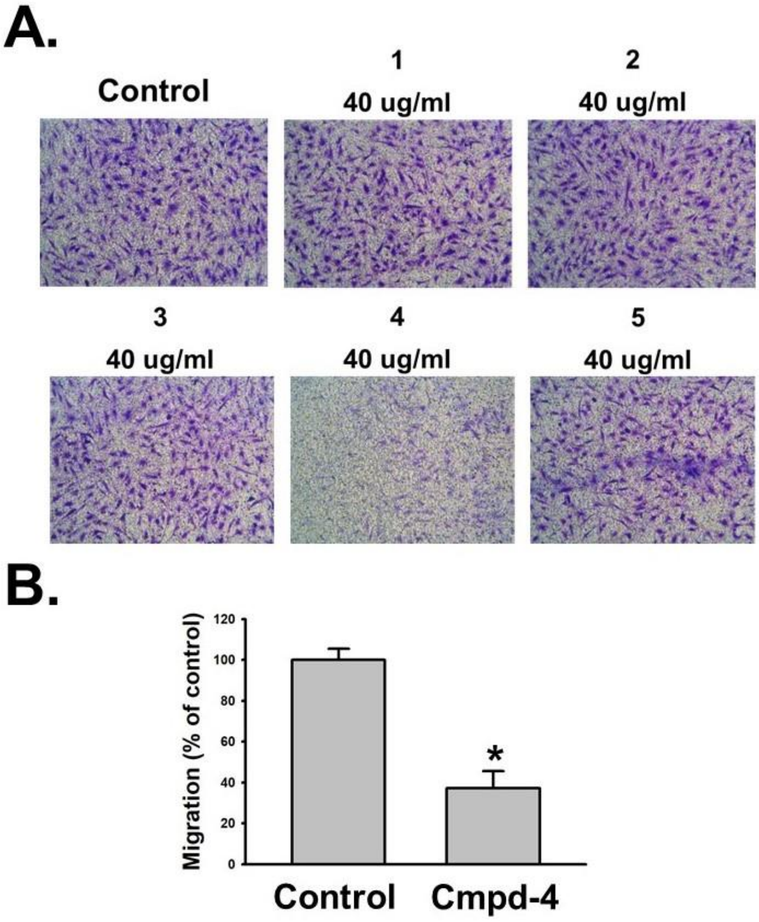

Figure S41. Effects of compounds 1-5 on cell migration of human endothelial progenitor cells. Cells were treated with the indicated compounds for 24 h. Cell migration was determined by Transwell migration assay. Data represent the mean ± S.E.M. of 3 independent experiments. *$p < 0.05$ compared with the control group.

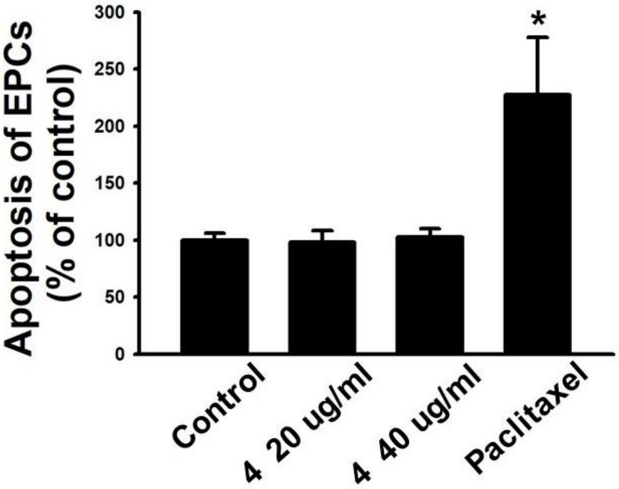

Figure S42. Effect of cmpd-4 on the apoptotic cell death of human endothelial progenitor cells. Cells were treated with the cmpd-4 and paclitaxel (10 μg/ml) for 24 h. Then, cell apoptosis was detected using the Cell Death ELISA^{PLUS} kit. Data represent the mean ± S.E.M. of 3 independent experiments. *p < 0.05 compared with the control group.

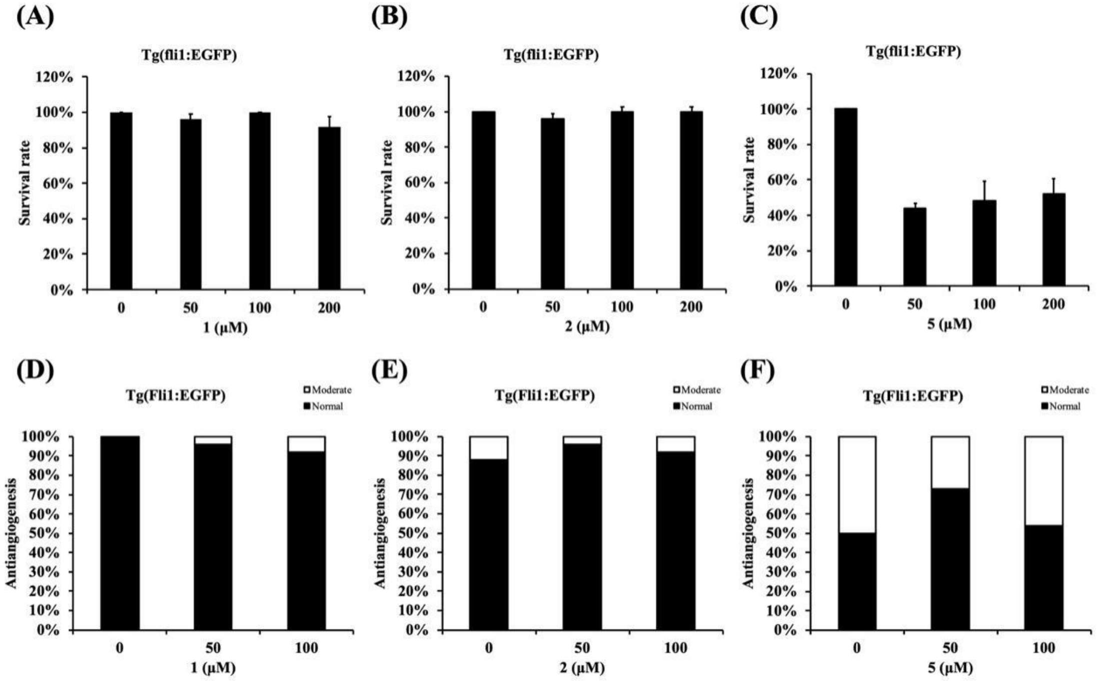

Figure S43. (A) Quantitative analysis presents percentage of fish embryos incubated with **1** with defective vasculature. $^{**}p < 0.01$ compared with the control group. (B) Quantitative analysis presents percentage of fish embryos incubated with **2** with defective vasculature. (C) Quantitative analysis presents percentage of fish embryos incubated with **5** with defective vasculature. (D) Quantitative analysis presents percentage of survival rate of fish embryos incubated with **1**. (E) Quantitative analysis presents percentage of survival rate of fish embryos incubated with **2**. (F) Quantitative analysis presents percentage of survival rate of fish embryos incubated with **5**.

```
                ....|....| ....|....| ....|....| ....|....| ....|....|
                        10         20         30         40         50
P.ITS           ACGTAACAAG GTTTCCGTAG GTGAACCTGC GGAAGAATCA TTACTGAGTG

                ....|....| ....|....| ....|....| ....|....| ....|....|
                        60         70         80         90        100
P.ITS           AGGGCCCCTC GGGGTCCAAC CTCCCACCCG TGTTTAACGA ACCTTTGTTG

                ....|....| ....|....| ....|....| ....|....| ....|....|
                       110        120        130        140        150
P.ITS           CTTCGGCGGG CCCGCCTCAC GGCCGCCGGG GGGCTCCTGC CCCCGGGCCC

                ....|....| ....|....| ....|....| ....|....| ....|....|
                       160        170        180        190        200
P.ITS           GCGCCCGCCG AAGCCCCCCC TTGAACGCTG TCTGAAGTTT GCAGTCTGAG

                ....|....| ....|....| ....|....| ....|....| ....|....|
                       210        220        230        240        250
P.ITS           AAACTAGCTA AATTAGTTAA AACTTTCAAC AACGGATCTC TTGGTTCCGG

                ....|....| ....|....| ....|....| ....|....| ....|....|
                       260        270        280        290        300
P.ITS           CATCGATGAA GAACGCAGCG AAATGCGATA ACTAATGTGA ATTGCAGAAT

                ....|....| ....|....| ....|....| ....|....| ....|....|
                       310        320        330        340        350
P.ITS           TCAGTGAATC ATCGAGTCTT TGAACGCACA TTGCGCCCTC TGGTATTCCG

                ....|....| ....|....| ....|....| ....|....| ....|....|
                       360        370        380        390        400
P.ITS           GAGGGCATGC CTGTCCGAGC GTCATTGCTG CCCTCAAGCA CGGCTTGTGT

                ....|....| ....|....| ....|....| ....|....| ....|....|
                       410        420        430        440        450
P.ITS           GTTGGGCCCC CGTCCCCCCC TCTGCCGGGG GGACGGGCCC GAAAGGCAGC

                ....|....| ....|....| ....|....| ....|....| ....|....|
                       460        470        480        490        500
P.ITS           GGCGGCACCG CGTCCGGTCC TCGAGCGTAT GGGGCTTCGT CACCCGCTCT

                ....|....| ....|....| ....|....| ....|....| ....|....|
                       510        520        530        540        550
P.ITS           TGTAGGCCCG GCCGGCGCCA GCCGACCCCA ACCCTAAATT TTTTTCAGGT

                ....|....| ....|....| ....|....| ....|....| ....|.
                       560        570        580        590
P.ITS           TGACCTCGGA TCAGGTAGGG ATACCCGCTG AACTTAAGCA TATCAA
```

Figure S44. ITS rDNA sequences.

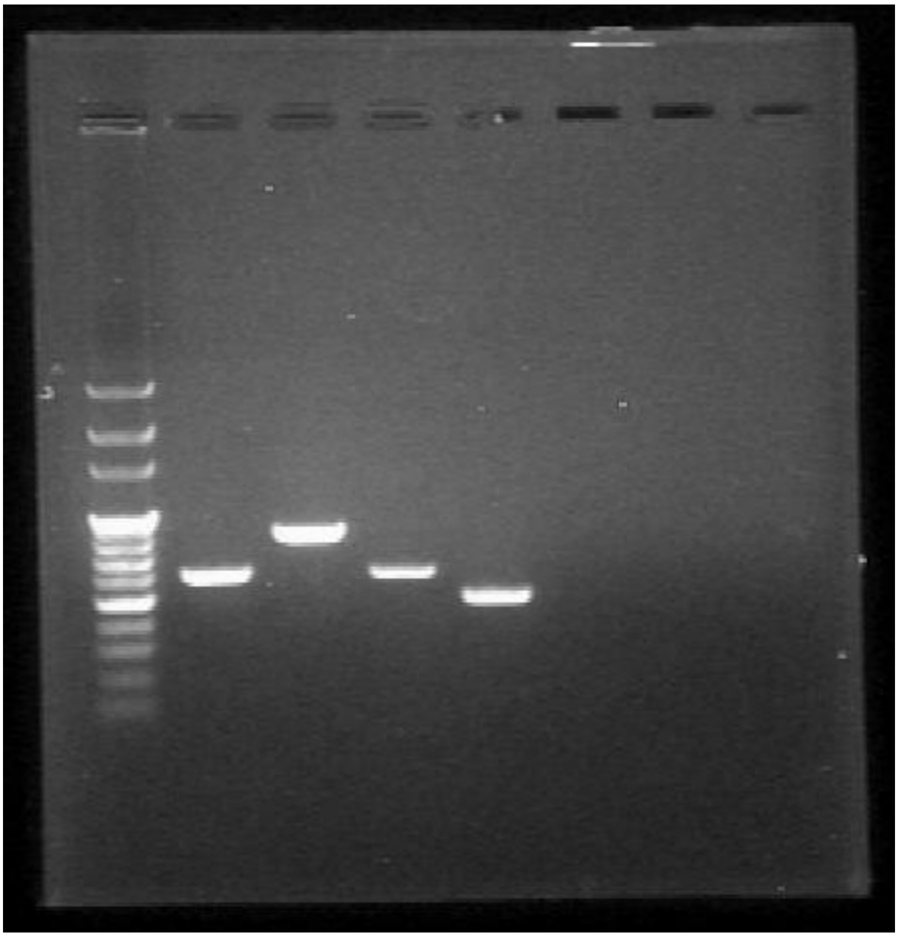

Figure S45. Agarose gel electrophoresis. Lane 1: DNA ladder. Lane 2: ITS rDNA. Lane 3: 28S rDNA. Lane 4: Calmodulin. Lane 5: Beta-tubulin

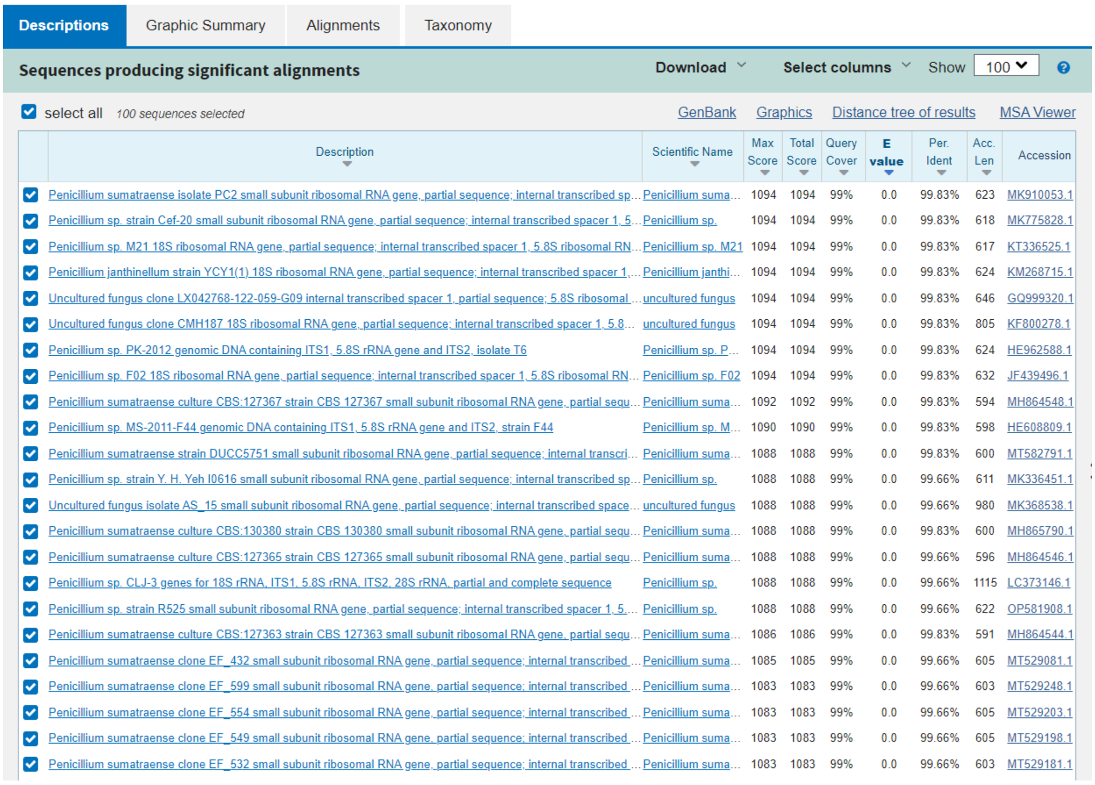

Descriptions | Graphic Summary | Alignments | Taxonomy

Sequences producing significant alignments — Download — Select columns — Show 100

select all 100 sequences selected — GenBank — Graphics — Distance tree of results — MSA Viewer

Description	Scientific Name	Max Score	Total Score	Query Cover	E value	Per. Ident	Acc. Len	Accession
Penicillium sumatraense isolate PC2 small subunit ribosomal RNA gene, partial sequence; internal transcribed sp...	Penicillium suma...	1094	1094	99%	0.0	99.83%	623	MK910053.1
Penicillium sp. strain Cef-20 small subunit ribosomal RNA gene, partial sequence; internal transcribed spacer 1, 5...	Penicillium sp.	1094	1094	99%	0.0	99.83%	618	MK775828.1
Penicillium sp. M21 18S ribosomal RNA gene, partial sequence; internal transcribed spacer 1, 5.8S ribosomal RN...	Penicillium sp. M21	1094	1094	99%	0.0	99.83%	617	KT336525.1
Penicillium janthinellum strain YCY1(1) 18S ribosomal RNA gene, partial sequence; internal transcribed spacer 1,...	Penicillium janthi...	1094	1094	99%	0.0	99.83%	624	KM268715.1
Uncultured fungus clone LX042768-122-059-G09 internal transcribed spacer 1, partial sequence; 5.8S ribosomal ...	uncultured fungus	1094	1094	99%	0.0	99.83%	646	GQ999320.1
Uncultured fungus clone CMH187 18S ribosomal RNA gene, partial sequence; internal transcribed spacer 1, 5.8...	uncultured fungus	1094	1094	99%	0.0	99.83%	805	KF800278.1
Penicillium sp. PK-2012 genomic DNA containing ITS1, 5.8S rRNA gene and ITS2, isolate T6	Penicillium sp. P...	1094	1094	99%	0.0	99.83%	624	HE962588.1
Penicillium sp. F02 18S ribosomal RNA gene, partial sequence; internal transcribed spacer 1, 5.8S ribosomal RN...	Penicillium sp. F02	1094	1094	99%	0.0	99.83%	632	JF439496.1
Penicillium sumatraense culture CBS:127367 strain CBS 127367 small subunit ribosomal RNA gene, partial sequ...	Penicillium suma...	1092	1092	99%	0.0	99.83%	594	MH864548.1
Penicillium sp. MS-2011-F44 genomic DNA containing ITS1, 5.8S rRNA gene and ITS2, strain F44	Penicillium sp. M...	1090	1090	99%	0.0	99.83%	598	HE608809.1
Penicillium sumatraense strain DUCC5751 small subunit ribosomal RNA gene, partial sequence; internal transcri...	Penicillium suma...	1088	1088	99%	0.0	99.83%	600	MT582791.1
Penicillium sp. strain Y. H. Yeh I0616 small subunit ribosomal RNA gene, partial sequence; internal transcribed sp...	Penicillium sp.	1088	1088	99%	0.0	99.66%	611	MK336451.1
Uncultured fungus isolate AS_15 small subunit ribosomal RNA gene, partial sequence; internal transcribed space...	uncultured fungus	1088	1088	99%	0.0	99.66%	980	MK368538.1
Penicillium sumatraense culture CBS:130380 strain CBS 130380 small subunit ribosomal RNA gene, partial sequ...	Penicillium suma...	1088	1088	99%	0.0	99.83%	600	MH865790.1
Penicillium sumatraense culture CBS:127365 strain CBS 127365 small subunit ribosomal RNA gene, partial sequ...	Penicillium suma...	1088	1088	99%	0.0	99.66%	596	MH864546.1
Penicillium sp. CLJ-3 genes for 18S rRNA, ITS1, 5.8S rRNA, ITS2, 28S rRNA, partial and complete sequence	Penicillium sp.	1088	1088	99%	0.0	99.66%	1115	LC373146.1
Penicillium sp. strain R525 small subunit ribosomal RNA gene, partial sequence; internal transcribed spacer 1, 5...	Penicillium sp.	1088	1088	99%	0.0	99.66%	622	OP581908.1
Penicillium sumatraense culture CBS:127363 strain CBS 127363 small subunit ribosomal RNA gene, partial sequ...	Penicillium suma...	1086	1086	99%	0.0	99.83%	591	MH864544.1
Penicillium sumatraense clone EF_432 small subunit ribosomal RNA gene, partial sequence; internal transcribed ...	Penicillium suma...	1085	1085	99%	0.0	99.66%	605	MT529081.1
Penicillium sumatraense clone EF_599 small subunit ribosomal RNA gene, partial sequence; internal transcribed ...	Penicillium suma...	1083	1083	99%	0.0	99.66%	603	MT529248.1
Penicillium sumatraense clone EF_554 small subunit ribosomal RNA gene, partial sequence; internal transcribed ...	Penicillium suma...	1083	1083	99%	0.0	99.66%	605	MT529203.1
Penicillium sumatraense clone EF_549 small subunit ribosomal RNA gene, partial sequence; internal transcribed ...	Penicillium suma...	1083	1083	99%	0.0	99.66%	605	MT529198.1
Penicillium sumatraense clone EF_532 small subunit ribosomal RNA gene, partial sequence; internal transcribed ...	Penicillium suma...	1083	1083	99%	0.0	99.66%	603	MT529181.1

Figure S46. BLASTn results of the isolated fungus.

Table S1. Crystal data and experimental details for **5**.

	Crystal data	
Empirical formula	C9 H14 O6	
Formula weight	218.20	
Crystal system	Orthorhombic	
Space group	$P2_12_12_1$	
Unit cell dimensions	a = 5.9346(2) Å	a= 90°.
	b = 10.4559(4) Å	b= 90°.
	c = 15.4630(6) Å	g = 90°.
Volume	959.50(6) $Å^3$	
Z	4	
F(000)	464	
Density (calculated)	1.510 Mg/m^3	
Wavelength	1.54178 Å	
Cell parameters reflections used	9848	
Theta range for Cell parameters	5.11 to 78.28°.	
Absorption coefficient	1.102 mm^{-1}	
Temperature	100(2) K	
Crystal size	0.300 x 0.150 x 0.100 mm^3	
	Data collection	
Diffractometer	Bruker D8 VENTURE	
Absorption correction	Semi-empirical from equivalents	
Max. and min. transmission	1.0000 and 0.8434	
No. of measured reflections	10303	
No. of independent reflections	1738 [R(int) = 0.0497]	
No. of observed [I>2_igma(I)]	1684	
Completeness to theta = 67.679°	99.8 %	
Theta range for data collection	5.106 to 67.995°.	
	Refinement	
Final R indices [I>2sigma(I)]	R1 = 0.0280, wR2 = 0.0795	
R indices (all data)	R1 = 0.0314, wR2 = 0.0814	
Goodness-of-fit on F^2	1.061	
No. of reflections	1738	
No. of parameters	142	
No. of restraints	0	
Absolute structure parameter	0.12(5)	
Largest diff. peak and hole	0.189 and -0.232 e.$Å^{-3}$	

Table S2. Atomic coordinates (x 10^4) and equivalent isotropic displacement parameters ($Å^2$x 10^3) For **5**. U(eq) is defined as one third of the trace of the orthogonalized U^{ij} tensor.

	x	y	z	U(eq)
O(1)	1009(2)	6114(1)	3996(1)	13(1)
O(2)	5513(2)	5513(1)	3423(1)	15(1)
O(3)	5830(2)	3279(1)	4317(1)	14(1)
O(4)	2023(2)	2828(1)	4119(1)	14(1)
O(5)	4135(2)	3855(1)	2640(1)	13(1)
O(6)	-256(2)	4631(1)	3046(1)	13(1)
C(1)	176(3)	3729(2)	2366(1)	22(1)
C(2)	733(3)	5337(2)	4769(1)	14(1)
C(3)	2932(3)	4639(2)	4968(1)	14(1)
C(4)	3693(3)	3812(2)	4215(1)	12(1)
C(5)	3785(3)	4635(2)	3388(1)	11(1)
C(6)	1614(3)	5415(2)	3264(1)	12(1)
C(7)	9(4)	6245(2)	5480(1)	15(1)
C(8)	2242(3)	2103(2)	3332(1)	14(1)
C(9)	2295(3)	2962(2)	2544(1)	16(1)

Table S3. Bond lengths [Å] and angles [°] for **5**.

O(1)-C(5)	1.395(2)
O(1)-C(1)	1.456(2)
O(2)-C(4)	1.394(2)
O(3)-C(3)	1.378(2)
O(4)-C(3)	1.420(2)
O(4)-C(7)	1.435(2)
O(5)-C(4)	1.430(2)
O(5)-C(8)	1.445(2)
O(6)-C(5)	1.436(2)
O(6)-C(9)	1.439(2)
C(1)-C(6)	1.513(2)
C(1)-C(2)	1.527(3)
C(2)-C(3)	1.519(3)
C(3)-C(4)	1.542(2)
C(4)-C(5)	1.537(2)
C(7)-C(8)	1.513(2)
C(8)-C(9)	1.517(3)
C(5)-O(1)-C(1)	113.76(13)
C(3)-O(4)-C(7)	114.44(14)
C(4)-O(5)-C(8)	110.00(13)
C(5)-O(6)-C(9)	113.72(14)
O(1)-C(1)-C(6)	106.15(14)
O(1)-C(1)-C(2)	109.64(15)
C(6)-C(1)-C(2)	113.34(15)
C(3)-C(2)-C(1)	111.83(15)
O(3)-C(3)-O(4)	110.68(14)
O(3)-C(3)-C(2)	114.26(15)
O(4)-C(3)-C(2)	106.39(14)
O(3)-C(3)-C(4)	106.53(14)
O(4)-C(3)-C(4)	109.79(14)
C(2)-C(3)-C(4)	109.17(14)
O(2)-C(4)-O(5)	107.66(13)
O(2)-C(4)-C(5)	105.96(13)
O(5)-C(4)-C(5)	108.86(14)
O(2)-C(4)-C(3)	111.48(14)

O(5)-C(4)-C(3)	110.93(13)
C(5)-C(4)-C(3)	111.74(14)
O(1)-C(5)-O(6)	107.10(14)
O(1)-C(5)-C(4)	113.12(14)
O(6)-C(5)-C(4)	112.24(13)
O(4)-C(7)-C(8)	111.24(14)
O(5)-C(8)-C(7)	108.42(14)
O(5)-C(8)-C(9)	107.67(13)
C(7)-C(8)-C(9)	116.28(16)
O(6)-C(9)-C(8)	111.74(14)

Symmetry transformations used to generate equivalent atoms:

Table S4. Anisotropic displacement parameters ($Å^2$x 10^3) for **5**. The anisotropic displacement factor exponent takes the form: $-2p^2[h^2 a^{*2}U^{11} + ... + 2 h k a^* b^* U^{12}]$

	U^{11}	U^{22}	U^{33}	U^{23}	U^{13}	U^{12}
O(1)	15(1)	13(1)	12(1)	-1(1)	0(1)	3(1)
O(2)	9(1)	14(1)	20(1)	3(1)	1(1)	0(1)
O(3)	12(1)	20(1)	14(1)	6(1)	-2(1)	4(1)
O(4)	15(1)	12(1)	11(1)	0(1)	0(1)	-3(1)
O(5)	14(1)	15(1)	11(1)	1(1)	3(1)	1(1)
O(6)	11(1)	16(1)	14(1)	-3(1)	-1(1)	0(1)
C(1)	15(1)	18(1)	11(1)	-1(1)	1(1)	1(1)
C(2)	16(1)	16(1)	10(1)	1(1)	-2(1)	-2(1)
C(3)	10(1)	12(1)	13(1)	2(1)	-2(1)	0(1)
C(4)	11(1)	12(1)	11(1)	0(1)	1(1)	1(1)
C(5)	11(1)	12(1)	12(1)	0(1)	0(1)	0(1)
C(6)	27(1)	25(1)	14(1)	-4(1)	3(1)	5(1)
C(7)	22(1)	12(1)	13(1)	-2(1)	0(1)	0(1)
C(8)	16(1)	14(1)	13(1)	-1(1)	-1(1)	0(1)
C(9)	17(1)	16(1)	12(1)	-3(1)	-4(1)	1(1)
